# Development and Validation of a Deep Learning Algorithm to Automatic Detection of Pituitary Microadenoma From MRI

**DOI:** 10.3389/fmed.2021.758690

**Published:** 2021-11-29

**Authors:** Qingling Li, Yanhua Zhu, Minglin Chen, Ruomi Guo, Qingyong Hu, Yaxin Lu, Zhenghui Deng, Songqing Deng, Tiecheng Zhang, Huiquan Wen, Rong Gao, Yuanpeng Nie, Haicheng Li, Jianning Chen, Guojun Shi, Jun Shen, Wai Wilson Cheung, Zifeng Liu, Yulan Guo, Yanming Chen

**Affiliations:** ^1^Department of Endocrinology and Metabolism, The Third Affiliated Hospital, Sun Yat-sen University, Guangzhou, China; ^2^Department of VIP Medical Service Center, The Third Affiliated Hospital, Sun Yat-sen University, Guangzhou, China; ^3^School of Electronics and Communication Engineering, Sun Yat-sen University, Guangzhou, China; ^4^Department of Radiology, The Third Affiliated Hospital, Sun Yat-sen University, Guangzhou, China; ^5^Department of Computer Science, University of Oxford, Oxfordshire, United Kingdom; ^6^Department of Medical Artificial Intelligence Center, The Third Affiliated Hospital, Sun Yat-sen University, Guangzhou, China; ^7^Department of Magnetic Resonance, The Second Affiliated Hospital, Harbin Medical University, Harbin, China; ^8^Department of Pathology, The Third Affiliated Hospital, Sun Yat-sen University, Guangzhou, China; ^9^Department of Radiology, The Sun Yat-sen Memorial Hospital, Sun Yat-sen University, Guangzhou, China; ^10^Department of Pediatrics, University of California, San Diego, San Diego, CA, United States

**Keywords:** pituitary microadenoma, magnetic resonance imaging, deep learning, algorithm, computer-aided diagnosis

## Abstract

**Background:** It is often difficult to diagnose pituitary microadenoma (PM) by MRI alone, due to its relatively small size, variable anatomical structure, complex clinical symptoms, and signs among individuals. We develop and validate a deep learning -based system to diagnose PM from MRI.

**Methods:** A total of 11,935 infertility participants were initially recruited for this project. After applying the exclusion criteria, 1,520 participants (556 PM patients and 964 controls subjects) were included for further stratified into 3 non-overlapping cohorts. The data used for the training set were derived from a retrospective study, and in the validation dataset, prospective temporal and geographical validation set were adopted. A total of 780 participants were used for training, 195 participants for testing, and 545 participants were used to validate the diagnosis performance. The PM-computer-aided diagnosis (PM-CAD) system consists of two parts: pituitary region detection and PM diagnosis. The diagnosis performance of the PM-CAD system was measured using the receiver operating characteristics (ROC) curve and area under the ROC curve (AUC), calibration curve, accuracy, sensitivity, specificity, positive predictive value (PPV), negative predictive value (NPV), and F1-score.

**Results:** Pituitary microadenoma-computer-aided diagnosis system showed 94.36% diagnostic accuracy and 98.13% AUC score in the testing dataset. We confirm the robustness and generalization of our PM-CAD system, the diagnostic accuracy in the internal dataset was 96.50% and in the external dataset was 92.26 and 92.36%, the AUC was 95.5, 94.7, and 93.7%, respectively. In human-computer competition, the diagnosis performance of our PM-CAD system was comparable to radiologists with >10 years of professional expertise (diagnosis accuracy of 94.0% vs. 95.0%, AUC of 95.6% vs. 95.0%). For the misdiagnosis cases from radiologists, our system showed a 100% accurate diagnosis. A browser-based software was designed to assist the PM diagnosis.

**Conclusions:** This is the first report showing that the PM-CAD system is a viable tool for detecting PM. Our results suggest that the PM-CAD system is applicable to radiology departments, especially in primary health care institutions.

## Introduction

A pituitary microadenoma (PM) is a tumor <10 mm in diameter. PMs can occur in either sex. As many as 10% of the population may have a microadenoma, but most do not cause symptoms (1, 2). However, some PMs cause symptoms by secreting hormones that exert harmful consequences, for example, in Cushing's disease, acromegaly, infertility, and hyperprolactinemia (1). Due to its small size and variable anatomical structure among individuals, the diagnosis of PM is not easy by applying the technique of MRI alone (3). Manual analysis of MRI data is usually biased and time-consuming, and the diagnostic accuracy is closely related to the experience of radiologists. A shortage of experienced radiologists may cause a delay in diagnosis and compromise the overall quality of service to patients with PM (4, 5). Deep learning has the potential to revolutionize disease diagnosis and management by improving the diagnostic accuracy of PM while reducing the workload of radiologists. The development of a convolutional neural network (CNN) has significantly improved the performance of image classification and object detection (6). Recent reports showed that a computer-aided diagnosis (CAD) system can accurately diagnose patients with pituitary adenoma from MR images (7–9). In this work, we have developed and validated an image-based deep learning model to aid the detection of PM.

## Materials and Methods

### Ethical Approval

This study is approved by the research ethics committee of the Institute of Basic Research in Clinical Medicine, The Third Affiliated Hospital of Sun Yat-sen University ([2020]02-089-01). This research is registered at the Chinese Clinical Trials Registry (http://www.chictr.org.cn/index.aspx) with the number ChiCTR2000032762.

### Data Collection and Pre-procession of MRI Data

The original intention to develop and validate the technique of deep learning algorithms assisting PM diagnosis was prompted by several misdiagnosed PM cases in our hospital (Supplementary Figure 1). We developed and validated an automatic diagnosis model for the detection of PM. The training set was a retrospective study, the data were extracted from January 2012 to September 2019 at The Third Affiliated Hospital of Sun Yat-sen University (TianHe and LuoGang hospital). The validation set 1 was a prospective temporal validation using data from October 2019 to April 2021 at The Third Affiliated Hospital of Sun Yat-sen University. Validation sets 2 and 3 are geographic prospective external validation with data from two additional hospitals (Sun Yat-sen Memorial Hospital of Sun Yat-sen University, and The Second Affiliated Hospital of Harbin Medical University) from March 2020 to April 2021. All data were recruited using the same inclusion and exclusion criteria.

**Figure 1:**
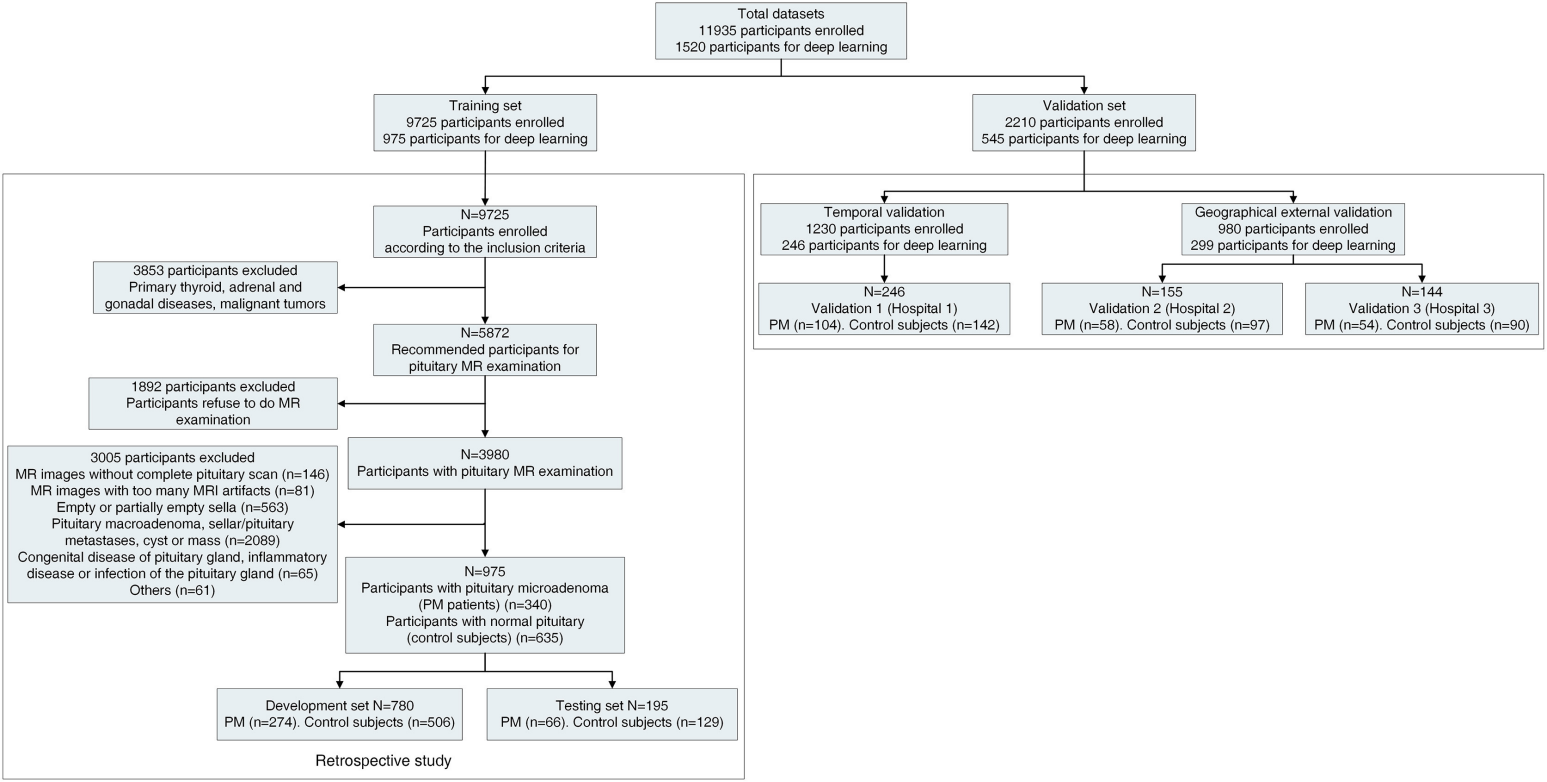
Workflow diagram for the overall experimental design. The detailed workflow diagram of the validation datasets are in Supplementary Figure 2. PM, pituitary microadenoma; MRI, magnetic resonance imaging. The Third Affiliated Hospital of Sun Yat-sen University as hospital 1. Sun Yat-sen Memorial Hospital of Sun Yat-sen University as hospital 2, and The Second Affiliated Hospital of Harbin Medical University as hospital 3.

The workflow diagram for the overall experimental design is in Figure 1 and Supplementary Figure 2. Inclusion criteria were participants suffered from infertility (defined as the inability of a sexually active couple to achieve pregnancy within a year or more with regular unprotected intercourse) and at least exhibited one or more of the following clinical symptoms/signs (menstrual irregularity, amenorrhea, galactorrhea, premature ejaculation, erectile dysfunction, or hypogonadism). Exclusion criteria were as follows: lactation, pregnancy, with primary thyroid, adrenal and/or gonadal diseases, malignant tumors, pituitary macroadenoma, sellar/pituitary masses or cyst, congenital disease of the pituitary gland, pituitaries, and MR images without complete pituitary scan or with too many MRI artifacts. Further examination was performed on the participants. We measured serum hormone levels of the participants (such as prolactin, adrenocorticotrophic hormone, follicle-stimulating hormone, luteinizing hormone, serum thyroid-stimulating hormone, and growth hormone) and performed a pituitary MR examination on those participants. Patients with functional and non-functional PM and patients with normal pituitary function were included for further deep learning analysis. The coronal dynamic enhancement T1-weighted imaging (T1WI) sequences of MRI (DICOM) from those participants were downloaded with a standard image format according to the software and instructions of the manufacturer. All pituitary images were read by two junior neuroradiologists (with <10 years of professional experience) and one senior neuroradiologist (with >10 years of professional experience), and the final diagnosis was mutually agreed upon by all three neuroradiologists have then proceeded for further investigation. In the training set, all images present with PM or normal pituitary images were selected by four general radiologists (>5 years of professional experience) and reviewed by two neuroradiologists (with >10 years of professional experience). All images of coronal dynamic enhancement T1WI sequence were used for the validation set without additional human intervention. MRI was performed with a 1.5 or 3.0 T MRI unit (GE, Philips company, Amsterdam, the Netherlands) in the head-first supine position, 380 ms/12.5 ms (repetition time /echo time), and 1 or 3 mm thick sections. Six medical fellows in the division of clinical endocrinology were involved in collecting patient clinical information, and the dataset was reviewed and verified by two endocrinologists.

### Model Structure (Overview of Our PM-CAD System)

The pipeline of our PM-CAD system is shown in Supplementary Figure 3, and it consists of two parts: (1) pituitary region detection and (2) PM diagnosis. All programs are implemented with Python (https://www.python.org/) language on PyTorch (https://pytorch.org/) platform. In pituitary region detection, we develop a pituitary detection model based on Faster R-CNN (10) [with ResNet-50 FPN (11) as its backbone]. The input MR image is processed by this model to generate classification and regression maps, which have been further used to extract the pituitary bounding box in MR images. The pituitary bounding box is used to crop the pituitary region patch from the MR image (Supplementary Method A). In PM diagnosis, we proposed a novel CNN (namely, PM-CAD) to diagnose the PM from the cropped MR images. All the cropped pituitary region images are resized to 256 × 256, normalized into (0,1), and processed with histogram matching normalization (HM) for the enhancement of microadenoma features. In the PM-CAD system, we modify the ResNet architecture to preserve fine-grained features during forward propagation. An attention module is used to further improve the discriminativeness of feature representation. To handle the overfitting problem, HM normalization, intensity shift data augmentation, and label-smoothing loss are used (Supplementary Method B). The training procedure is stopped after 500 epochs (iterations through the entire dataset) due to the absence of further improvement in terms of both the area under receiver operating curve (AUC) and label-smoothing loss (Supplementary Figure 4).

### Model Discrimination and Calibration

A total of 1,520 participants were included for the further study. We partitioned the data into three non-overlapping sets, with 780 participants for model development, 195 participants for model testing (developing and testing dataset as 8:2), and 545 participants for model validation. To reduce the time bias, the training set was a retrospective study from January 2012 to September 2019. The validation set was a prospective validation from October 2019 to April 2021. The detailed statistics for each set are summarized in Figure 1 and Supplementary Figure 2.

### Evaluation of the Diagnosis Performance of Our PM-CAD System and Statistical Analysis

In the testing set, we used accuracy, sensitivity, specificity, positive predictive value (PPV), negative predictive value (NPV), and F1-score to evaluate our PM-CAD system. The validation set A had been used to evaluate the generalization ability and stability of our PM-CAD system. The receiver operating characteristics (ROC; showing both true-positive rate and false-positive rate for diagnosis performance) curves and AUC were used in testing, internal and external validation sets (12, 13). We also used binary logistic regression methods to re-fit the prediction probability data rooted in PM-CAD, and calibration curves were used to test the fitting ability of the model (14). Validation set B consists of 100 participants and has been used to compare the performance of the PM-CAD system to general radiologists. A wide range of performance metrics has been adopted, such as diagnosis accuracy, sensitivity, specificity, PPV, NPV, F1-score, weighted error, positive likelihood ratio (PLR), negative likelihood ratio (NLR), and AUC (12). A weighted error was used for further analysis, specifically, a penalty weight of 2 was assigned to false-negative cases and a penalty weight of 1 was assigned to false-positive cases (12). Six radiologists were recruited for this study. Radiologists 1 and 2 have professional experience of <5 years, Radiologists 3 and 4 have professional experience between 5 and 10 years, and Radiologist 5 and 6 have professional experience over 10 years. Each radiologist read MR images of 100 participants independently. The Bland-Altman plot was used to evaluate the interobserver consistency of pituitary MRI finding independently measured by the six radiologists. The diagnostic accuracy of those radiologists was evaluated, and the experience of each radiologist in reading images of the cranial and pituitary MR or CT is shown in Supplementary Table 1. In validation set C, we tested the diagnosis accuracy of our PM-CAD system on three cases misdiagnosed by radiologists. Descriptive statistics included mean (SD) for continuous variables and proportions for categorical variables. All the metrics were calculated using Python-3.9.5 (https://www.python.org/), and R-4.0.3 (15) was used to provide visual analyses.

### Browser-Based Software Application

A browser-based software was designed to assist the diagnosis from pituitary MR images. Once pituitary MR images (DICOM files) are uploaded to the software, PM diagnosis outputs can be presented.

## Results

### Study Participants

A total of 11,935 infertility participants were initially recruited for this project. After applying the exclusion criteria, 1,520 participants (556 PM patients and 964 controls subjects) were included for further study whereby we have partitioned data from 975 participants (340 PM patients and 635 control subjects) for the training set, such as 780 participants (19,573 images) for development set and 195 participants (4,927 images) for the testing set. In the validation set, 545 participants (13,239 images) were recruited for the study. The validation set A consisted of 163 PM patients and 279 control subjects came from three hospitals. The validation set B consisted of 100 participants (50 PM patients, and 50 control subjects). In validation set C, we tested the diagnosis accuracy of our PM-CAD system on three misdiagnosed PM cases. The detailed statistics for each set are summarized in Figure 1 and Supplementary Figure 2. Among patients with PM, there were 397 cases of non-functional PMs and 159 cases of functional PMs. The clinical and baseline characteristics of these participants are shown in Table 1.

**Table 1:** Description and characteristics of the training and validation datasets.

**Characteristics**	**Training set**				**Validation set**					
	**Development set**		**Testing set**		**Temporal validation** **(hospital 1)**		**Geographical validation** **(hospital 2)**		**Geographical validation** **(hospital 3)**	
	**Patients**	**Controls**	**Patients**	**Controls**	**Patients**	**Controls**	**Patients**	**Controls**	**Patients**	**Controls**
**Full cohort**	274	506	66	129	104	142	58	97	54	90
Sex [No. (%)]										
Male	56 (20.4)	98 (19.4)	13 (19.7)	30 (23.3)	19 (18.3)	25 (17.6)	12 (20.7)	22 (22.7)	10 (18.5)	17 (18.9)
Female	218 (79.6)	408 (80.6)	53 (80.3)	99 (76.7)	85 (81.7)	117 (82.4)	46 (79.3)	75 (77.3)	44 (81.5)	73 (81.1)
Age (Mean ± SD)	30.92 ± 6.56	31.26 ± 7.36	30.82 ± 6.02	30.58 ± 6.04	30.79 ± 6.86	30.66 ± 5.50	31.43 ± 7.61	30.86 ± 5.46	29.81 ± 4.78	30.14 ± 5.35
BMI (Mean ± SD)	23.07 ± 2.50	23.09 ± 2.52	22.85 ± 2.38	23.91 ± 2.48	23.20 ± 2.40	22.67 ± 2.31	23.42 ± 2.79	23.21 ± 2.57	23.27 ± 2.50	23.48 ± 2.66
**Blood biochemical indices (Mean ± SD)**										
PRL, uIU/mL	1,184.92 ± 1,353.99	321.14 ± 144.32	1,142.82 ± 1,332.77	302.21 ± 150.47	1,121.06 ± 1,362.23	301.31 ± 152.69	1,053.70 ± 1,346.33	329.89 ± 149.50	1,150.89 ± 1,280.17	304.69 ± 162.74
ACTH, pmol/L	5.69 ± 2.46	5.61 ± 1.80	5.48 ± 3.57	5.34 ± 1.82	5.91 ± 4.06	5.29 ± 2.03	5.40 ± 1.71	5.17 ± 1.69	5.35 ± 1.54	5.12 ± 1.70
FSH, mIU/mL	4.73 ± 2.32	4.72 ± 2.04	5.02 ± 2.27	4.53 ± 2.01	5.17 ± 1.94	4.47 ± 2.26	5.53 ± 2.10	4.58 ± 2.07	5.14 ± 2.26	4.49 ± 2.00
LH, mIU/mL	4.24 ± 2.02	4.39 ± 1.93	4.34 ± 2.32	4.32 ± 1.82	4.97 ± 2.22	4.12 ± 1.98	5.80 ± 2.13	4.34 ± 1.75	4.79 ± 1.84	4.38 ± 1.79
TSH, uIU/mL	2.07 ± 0.93	2.48 ± 1.21	2.10 ± 0.89	2.19 ± 1.08	2.02 ± 0.81	1.92 ± 0.84	1.99 ± 1.47	1.96 ± 0.85	1.90 ± 0.79	2.08 ± 0.87
**MRI examination and PM Functional diagnosis [No. (%)]**										
Normal pituitary of MRI scan	–	506	–	129	–	142	–	97	–	90
PM of MRI scan	274	–	66	–	104	–	58	–	54	–
Non-functional PM	194 (70.8)	–	47 (71.2)	–	75 (72.1)	–	42 (72.4)	–	39 (72.2)	–
Functional PM	80 (29.2)	–	19 (28.8)	–	29 (27.9)	–	16 (27.6)	–	15 (27.8)	–
PRL-PM	76	–	17	–	24	–	15	–	15	–
ACTH-PM	3	–	2	–	3	–	0	–	0	–
GH-PM	1	–	0	–	2	–	0	–	0	–
TSH-PM	0	–	0	–	0	–	1	–	0	–

*Data are mean (S.D.) or a number of individuals (%). BMI, Body Mass Index; PRL, Prolactin; ACTH, adrenocorticotrophic hormone; FSH, Follicle-Stimulating Hormone; LH, Luteinizing Hormone; TSH, Serum Thyroid-stimulating Hormone; GH, Growth hormone; MRI, Magnetic Resonance Imaging; PM, pituitary microadenoma.—means the participants did not calculate*.

### Performance of PM-CAD System

**Figure 2:**
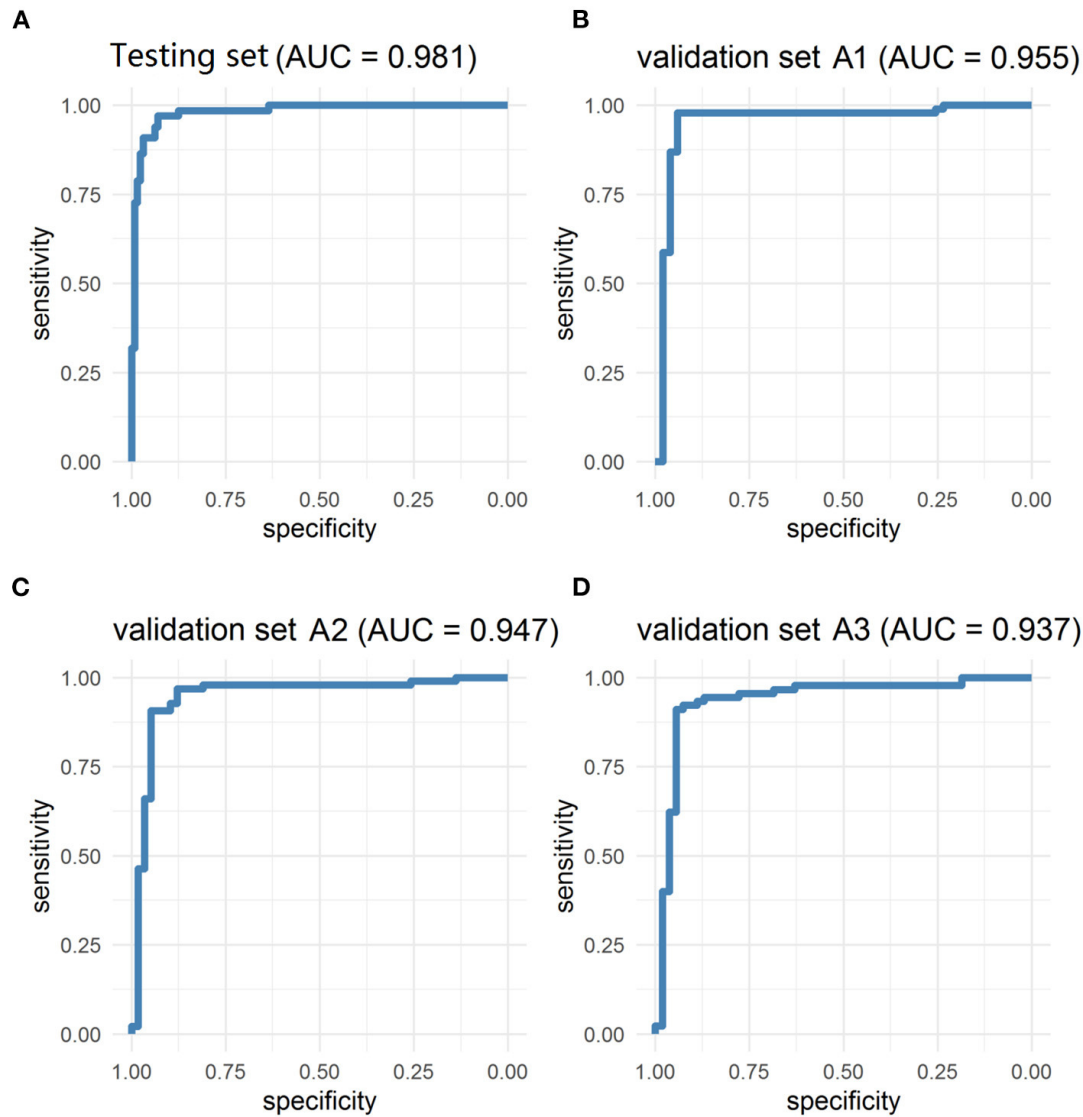
The ROC curves of testing and validation set A1 (Internal dataset), validation set A2 and A3 (external dataset). The model has achieved excellent diagnosis performance in internal and external data sets. **(A)** The AUC of the testing set was 98.13%. **(B)** The validation set A1 is a temporal internal dataset, the AUC was 95.46%. **(C,D)** In the geographical external dataset, the AUC of the validation set A2 and A3 was 94.72 and 93.70%, respectively. AUC, area under the ROC curve; ROC, the receiver operator curve.

**Figure 3:**
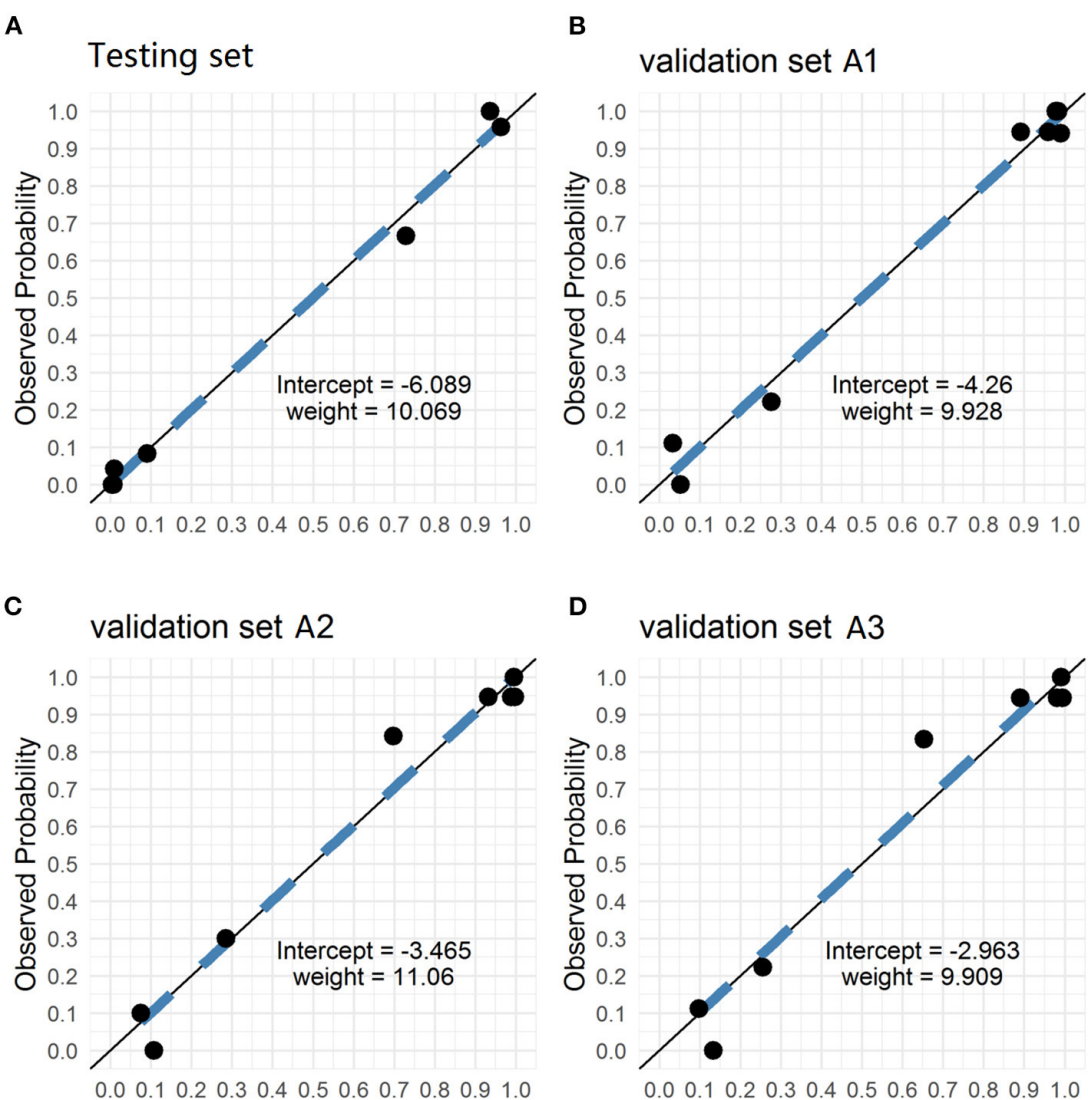
The Calibration curves of testing and validation set A1 (Internal dataset), validation set A2 and A3 (external dataset). The calibration curves of the predicted probability from our PM-CAD vs. the observed probability for PM in **(A)** the testing set, **(B)** the validation set A1, **(C)** the validation set A2, and **(D)** the validation set A3. We used logistic regression to rebuild the prediction probability from our CNN model. The intercepts on the testing and verification set A are −6.098, −4.26, −3.465, and −2.963, respectively. And the probability weight W is 10.069, 9.928, 11.06, and 9.909, respectively. CNN, convolutional neural network; PM-CAD, Pituitary microadenoma-computer-aided diagnosis.

The PM-CAD system consists of two parts: pituitary region detection and PM diagnosis. In pituitary region detection, we use the well-known average precision (AP) as the evaluation metric. We achieved an AP of 0.9783 at an intersection-of-union (IOU) threshold of 0.5 (Supplementary Method A). For testing the accuracy of PM diagnosis, 975 participants have been used for the development and testing set (Supplementary Method B). We showed that our PM-CAD system achieved an AUC of 98.13% (Figure 2A), an F1-score of 92.09%, an accuracy of 94.36%, a sensitivity of 96.97%, a PPV of 87.67%, a specificity of 93.02%, and an NPV of 98.36% on the testing set. The calibration curve of the testing set is listed in Figure 3A, the intercept on the testing is −6.098, and the probability weight W is 10.069. We employed PM-CAD for further investigation.

### PM-CAD System Application in the Validation Set (Internal and External Datasets)

We used the internal and external datasets to validate the robust generalization performance of our PM-CAD system. The system was further tested in 442 participants from three different hospitals (Validation set A). The PM-CAD system achieved the diagnosis performance of AUC (95.46%) (Figure 2B), F1-score (97.30%), accuracy (96.50%), sensitivity (97.83%), PPV (96.77%), specificity (94.12%), and NPV (96.00%) in hospital 1. In hospital 2, the AUC is 94.72% (Figure 2C), F1-score is 93.62%, accuracy is 92.26%, sensitivity is 90.72%, PPV is 96.70%, specificity is 94.83%, and NPV is 85.94%, respectively. The diagnosis performance is AUC (93.70%) (Figure 2D), F1-score (93.71%), accuracy (92.36%), sensitivity (91.11%), PPV (96.47%), specificity (94.44%), and NPV is (86.44%) in hospital 3 (Table 2). The ROC curve is described in Figures 2B–D. The calibration curve of the validation set A is in Figures 3B–D, the intercept is −4.26, −3.465, and −2.963, respectively. And the probability weight W was 9.928, 11.06, and 9.909, respectively. The classification confusion matrices report the number of true positive, false positive, true negative, and false negative, which are resulted in Supplementary Table 2. We showed that our PM-CAD system achieves excellent diagnostic performance in internal and external datasets.

**Table 2:** The diagnosis performance of the PM-CAD system in the validation set A (internal and external datasets).

**Evaluation metrics**	**Validation set A** **(set A1: internal dataset, hospital 1)**	**Validation set A** **(set A2: external dataset, hospital 2)**	**Validation set A** **(set A3: external dataset, hospital 3)**
AUC (95% CI)	0.9546 (0.9028–0.9923)	0.9472 (0.8978–0.9858)	0.9370 (0.8821–0.9802)
Sensitivity	0.9783 (0.9237–0.9974)	0.9072 (0.8312–0.9567)	0.9111 (0.8324–0.9608)
Specificity	0.9412 (0.8376–0.9877)	0.9483 (0.8562–0.9892)	0.9444 (0.8461–0.9884)
Accuracy	0.9650 (0.9203–0.9885)	0.9226 (0.8687–0.9594)	0.9236 (0.8674–0.9613)
PPV	0.9677 (0.9086–0.9933)	0.9670 (0.9067–0.9931)	0.9647 (0.9003–0.9927)
NPV	0.9600 (0.8629–0.9951)	0.8594 (0.7498–0.9336)	0.8644 (0.7502–0.9396)
F1 score	0.9730 (0.9381–0.9912)	0.9362 (0.8912–0.9666)	0.9371 (0.8903–0.9682)

*AUC, the area under the receiver operating characteristic curve; PPV, positive predictive value; NPV, negative predictive value*.

### Performance of the PM-CAD System vs. Radiologists

An independent validation set B (100 participants: 50 PM patients and 50 controls from hospital 1) was used to compare the performance of the PM-CAD system vs. radiologists. For this comparison, six radiologists were recruited. The diagnosis performance of PM-CAD system is F1-score (93.88%), accuracy (94.00%), sensitivity (92.00%), PPV (95.83%), specificity (96.00%), and NPV is (92.31%) (Supplementary Table 3). In contrast, the performance of our best radiologist #6 is F1-score (94.95%), accuracy (95.00%), sensitivity (94.00%), PPV (95.92%), specificity (96.00%), and NPV is (94.12%) (Supplementary Table 3). The ROC curves are shown in Supplementary Figure 5A, the AUC of the PM-CAD system was 95.56% and outperformed our six radiologists (best radiologist #6 as 95.00%), at the same false-positive rate, the true positive rate of the PM-CAD system was higher than six radiologists (Supplementary Figure 5A). Weighted error scoring (10) was incorporated during modeling and evaluation, the PM-CAD system produces a weighted error of 10.00%, which is far below the average weighted error of 21.67% achieved by six radiologists (Supplementary Figure 5B). The difference of NLRs or PLRs (10) between our PM-CAD system and radiologists is shown in Supplementary Figures 5C,D, our model demonstrates excellent diagnostic performance. The classification confusion matrices report the number of true positive, false positive, true negative, and false negative resulted for the PM-CAD system and radiologists in Supplementary Table 4. Thus, we showed that the diagnosis performance of our PM-CAD system is comparable to general radiologists with more than 10 years of professional experience. A Bland-Altman plot was used to analyze the interobserver consistency of the six radiologists' independent measurements of the pituitary MRI finding. The 95% limits of agreement were −0.4500 to 0.4300, −0.2958 to 0.2558, −0.1860 to 0.2060, −0.1860 to 0.2060, −0.1860 to 0.2060, and −0.2060 to 0.1860, respectively, indicating high interobserver consistency.

### Further Assessment for the Diagnosis Performance of the PM-CAD System

We sampled three double positive cases of PM (both diagnosed by radiologists and PM-CAD system), which underwent surgical treatment, the double positive cases were confirmed by a subsequent pathological examination (one case of Cushing's disease, one case of Acromegalia, and one case of prolactinoma; Supplementary Figure 6A).

A false-negative diagnosis leads to delay in treatment of PM, PM-CAD system showed 100% diagnosis accuracy of detecting three clinically misdiagnosed PM cases which subsequently underwent surgical treatment (two cases of Cushing's disease and one case of thyroid-stimulating hormone, TSH, secreting PM; Supplementary Figure 6B). The diagnosis of thE misdiagnosed PM was confirmed by histopathology examination and relevant clinical information (Supplementary Figure 6 and Supplementary Table 5).

### Browser-Based Software Application

The browser-based software was designed to assist the PM diagnosis of pituitary MR images from different hospitals, which is hosted at http://www.pituitarymicroadenoma.com. Even without graphics processing unit (GPU) acceleration, the application takes only 1–2 s to analyze all MR images from a patient. Once DICOM files (the coronal dynamic enhancement T1-weighted imaging (T1W) sequence) are uploaded to the software, PM diagnosis outputs can be presented. The software interface is presented in Supplementary Figure 7. In a prospective study, we have tested the efficacies of our PM-CAD in the division of endocrinology in our hospital. Our results indicate that the PM-CAD system is an excellent screening test for the presence of PM. Over a period of 1 month, our PM-CAD system was able to detect the presence of 11 PM patients with a 97% accuracy rate (of 48 infertile patients and 25 patients with pituitary MR examination).

## Discussion

In this work, we developed a deep learning system (namely, PM-CAD) to diagnose PM from MRI. As we know, it is the first attempt to focus on PM diagnosis by using deep learning, although similar works have been proposed for pituitary adenoma (7–9, 16). Diagnosis of PM is challenging due to its tiny size and various anatomical structure (1–3). We found that our PM-CAD system can accurately diagnose PM from MRI without additional information, the system achieves a 96.5% diagnostic accuracy, which is comparable to radiologists with over 10 years of professional expertise.

Several previous works have attempted to analyze pituitary adenoma using MRI. Ugga et al. (9) used a machine learning method to extract MRI-based radiomics to predict the proliferative index of pituitary macroadenomas. Qian et al. (7) employ a CNN network to diagnose pituitary adenoma from MRI, they evaluated a 149 participants dataset, which includes pituitary macroadenoma and microadenoma. Wang et al. (16) created an automated segmentation method for the sellar region, several tools to extract invasiveness-related features of pituitary adenoma and evaluate their clinical usefulness by predicting the tumor consistency. In this study, we focus on the diagnose of PM from the PM-CAD system with a large dataset. We show that our PM-CAD system outperforms the model developed by Qian et al. (7). Because of our PM-CAD system can specifically extract PM features from pituitary MR images and trained with more data. In addition, our model was validated in three hospitals and showed excellent generalization ability.

### Strengths and Limitations

Our work has the following strengths. First, we showed that this PM-CAD system is a rapid, reliable tool to diagnose PM with a high accuracy in both internal and external datasets. Second, PM diagnosis requires experienced radiologists, but the exhausting workload raises the misdiagnose rate. Our PM-CAD system can be used as an assistant tool to reduce the workload of radiologists. Our PM-CAD system achieves comparable diagnostic accuracy to experienced radiologists and can make a decision in 1–2 s. Third, medical resources are not evenly distributed, that is, experienced radiologists mostly worked in economically developed areas hospitals while economically underdeveloped areas are lack experienced radiologists (4, 5). Our online accessible PM-CAD system can provide PM diagnosis to these areas and improve their PM diagnostic capabilities. Last, training a radiologist is costly and time consuming. It usually takes more than 10 years to train a qualified radiologist (4, 5). Our PM-CAD system is trained from annotated data and takes few time (about 30 s per patient) to improve its performance when more data are provided.

Our PM-CAD system remains several problems to be solved. First, although our PM-CAD system achieves a 96.5% diagnostic accuracy, this implies that 3.5% of cases may potentially be misdiagnosed in practice. To further improve the diagnosis performance of the PM-CAD system, more data should be collected and used to train our models. Second, when more new data are available, it would be better than our PM-CAD system can perform model self-update, a continual learning approach can be introduced to keep our system learning. Third, MRI scan data are unique to patients, with privacy concerns, these data are not allowed to distribute out of the hospitals. Therefore, our PM-CAD system cannot be fine-tuned in a specific hospital. In future work, we will use a federated learning framework to fine-tune our models in a privacy-preserving manner.

## Conclusions

In summary, we have developed a deep learning-based system (namely, PM-CAD) to detect PM from MRI. A Total of 1,520 participants datasets have been used to train, validate, and test our system. Our PM-CAD system achieves a diagnostic accuracy comparable to radiologists with over 10 years of professional expertise. In the study, our PM-CAD system shows excellent generalization ability. Results from this work highlight the potential applications of deep learning on the diagnosis of patients with PM. With the rapid development of computing power, deep learning algorithms can surpass the gold diagnosis standard for the detection of PM. Machine learning for the diagnosis of PM will serve as an important component in improving patient care and outcomes.

## Data Availability Statement

The original contributions presented in the study are included in the article/Supplementary Material, further inquiries can be directed to the corresponding author/s.

## Code Availability Statement

The software and code of the proposed method have been separated into two files and are available as Supplementary Software files. https://github.com/MinglinChen94/PituitaryMicroadenomaDiagnosis.

## Ethics Statement

The studies involving human participants were reviewed and approved by the Research Ethics Committee of the Institute of Basic Research in Clinical Medicine, The Third Affiliated Hospital of Sun Yat-sen University ([2020]02-089-01). This research is registered at the Chinese Clinical Trials Registry (http://www.chictr.org.cn/index.aspx) with the number ChiCTR2000032762. The patients/participants provided their written informed consent to participate in this study.

## Author Contributions

YC and YG: have full access to all the data in the study, take responsibility for the integrity of the data and the accuracy of the data analysis, administrative, technical, material support, and supervision. QL, YZ, MC, ZL, and GS: concept and design. QL and MC: drafting of the manuscript. WC and GS: critical revision of the manuscript for important intellectual content. ZL, MC, and QL: statistical analysis. YC, YG, and RGu: obtained funding. All authors acquisition, analysis, or interpretation of data.

## Funding

This study was funded by the National Key R&D Program of China (2017YFA0105803), the National Natural Science Foundation of China (U20A20185, 81770826, 61972435, and 81801757), the Key Area R&D Program of Guangdong Province (2019B020227003), the Natural Science Foundation of Guangdong Province (2019A1515011271, 2019A1515012051, and 2018A030310322), the Science and Technology Plan Projects of Guangzhou (202007040003), and the Science and Technology Innovation Committee of Shenzhen Municipality (JCYJ20190807152209394).

## Conflict of Interest

The authors declare that the research was conducted in the absence of any commercial or financial relationships that could be construed as a potential conflict of interest.

## Publisher's Note

All claims expressed in this article are solely those of the authors and do not necessarily represent those of their affiliated organizations, or those of the publisher, the editors and the reviewers. Any product that may be evaluated in this article, or claim that may be made by its manufacturer, is not guaranteed or endorsed by the publisher.

## Supplementary Material

Supplementary Method A detailed description of PM-CAD Model. PM-CAD, Pituitary microadenoma-computer-aided diagnosis.

Supplementary Figure 1 Cases of 4 misdiagnosed pituitary microadenomas. **(A)** 4 consecutive pituitary MRI scans over a period of 20 months in a misdiagnosed patient with pituitary microadenoma. The radiologists have not detected the pituitary microadenoma during the first 3 MRI examinations. A functional microadenoma has been localized by the subsequent ACTH examination of the inferior petrosal sinus in the region of right pituitary gland. On the 4th MRI scanning, two microadenoma are detected by radiologist. **(B)** Additional 3 cases of misdiagnosed microadenoma. Patient 1 has a very small microadenoma with a diameter <3 mm. Patient 2 has an irregularly shaped microadenoma. Patient 3 has two microadenoma (with diameters of 2.8 mm and 6.1 mm, respectively) and the smaller one was misdiagnosed. The comprehensive clinical data for patients were listed in Supplementary Table 1. ACTH, Adrenocorticotropic Hormone; MRI, magnetic resonance imaging; T1WI-COR, T1 weighted imaging-coronal. MRI bar = 5 mm. The yellow arrow and the area inside the red circle represent adenomas.

Supplementary Figure 2 Workflow diagram for the validation datasets. PM, pituitary microadenoma; MRI, magnetic resonance imaging.

Supplementary Figure 3 Overview of our PM-CAD system. **(A)** First the MR images are fed into our PM-CAD system for automatic diagnosis. The proposed PM-CAD system consists of two models: **(B)** the pituitary detection model localizes the pituitary region in cerebral MRI. The MR images are processed with multiple convolutional layers and two maps (classification map is used to predict the center and the regression map is used to refine the height and width of the rectangle box) are produced to predict a rectangle box enclosing the pituitary region. The pituitary rectangle region is cropped, stacked, and then fed into the PM diagnosis model. **(C)** It employs the proposed PM-CAD model to extract features. A softmax layer is employed to transform the feature into the presence probability of PM. CAD, computer-aided diagnosis; MRI, magnetic resonance imaging; MR, magnetic resonance; PM, pituitary microadenoma.

Supplementary Figure 4 Performance of the PM-CAD system on the training datasets. **(A)** Accuracy curves achieved by the PM-CAD system on the development and testing datasets. **(B)** Cross entropy loss curves achieved by the PM-CAD system on the development and testing datasets. We train the PM-CAD system for 500 epochs.

Supplementary Figure 5 The PM-CAD system outperforms 6 radiologists in AUC of PM diagnosis. **(A)** ROC and AUC: ROC curve shows the true positive rates (sensitivity) with respect to different false-positive rates (1-specificity). The ROC curve shows that the PM-CAD system outperforms 6 radiologists. The AUC of PM-CAD system is 95.6% better than our best radiologist#6 (AUC 95.0%). **(B)** Weighted error. A penalty weight of 2 is applied to false-negatives and a penalty weight of 1 is assigned to false-positives. The PM-CAD system produces a weighted error of 10%, whereas the radiologists produce a weighted error of 21.67%. **(C,D)** The negative likelihood ratio and the positive likelihood ratio: The negative likelihood ratio is defined as the false-negative rate over the true negative rate, so that a decreasing likelihood ratio <1 indicated increasing probability the absence of PM. The positive likelihood ratio is defined as the true positive rate over the false-positive rate, so that an increasing likelihood ratio > 1 indicated increasing probability the diagnosis of PM. The confidence intervals show that the PM-CAD system demonstrates statistically better screening performance in terms of both negative likelihood ratio and positive likelihood ratio than radiologists. Radiologist 1 & 2: with < 5 years professional experience, Radiologist 3 & 4: with 5 - 10 years professional experience, Radiologist 5 & 6: with > 10 years professional experience. PM, pituitary microadenoma; receiver operating characteristics (ROC); the area under ROC curve (AUC).

Supplementary Figure 6 The MRI and histological validation of double positive and false-negative cases. **(A,B)** 3 double positive and 3 false-negative cases, which were functional PM, as confirmed by subsequent pathological examination. The comprehensive clinical data for these patients are listed in Supplementary Table 5. PM, pituitary microadenoma; MRI, magnetic resonance imaging; AI, Artificial intelligence; HE, hematoxylin and eosin; ACTH, adrenocorticotropic hormone; GH, growth hormone; TSH, thyroid stimulating hormone; PRL, prolactin. MR bar = 5mm. Pathology bar =100 μm. The yellow arrow indicates a pituitary microadenoma.

Supplementary Figure 7 The browser-based software to aid the diagnosis of PM. As long as we upload the pituitary MR images (DICOM), the software will tell you whether the patient suffering from PM disease. This browser based tool can be accessed at http://82.157.181.77/.

Supplementary Table 1 The workload of radiologists with different professional experience in human-computer competition. All participating radiologists are general radiologists (no specialization). Workload analysis was performed on the participating radiologists for 1 year.

Supplementary Table 2 Confusion Matrices for testing and validation of dataset A (internal and external datasets). Data are numbers of images. a, true-positive; b, false-positive; c, false-negative; d, true-negative.

Supplementary Table 3 The diagnostic performance for Human-computer competition according to temporal validation set B (*n* = 100). Unless otherwise specified, data are percentages, with numbers of images in parentheses and 95% confidence intervals in brackets. F1 score, the harmonic mean of PPV and sensitivity. NPV, negative predictive value; PPV, positive predictive value. Radiologist 1 & 2, < 5 years professional experience; Radiologist 3 & 4, 5 - 10 years professional experience; Radiologist 5 & 6, > 10 years professional experience.

Supplementary Table 4 Confusion Matrices for Human-computer competition according to temporal validation set B (*n* = 100). Data are numbers of images. a, true-positive; b, false-positive; c, false-negative; d, true-negative.

Supplementary Table 5 The patient clinical data in Supplementary Figures 1, 6. PM, pituitary microadenoma; BMI, Body Mass Index; SBP, Systolic Blood Pressure; DBP, Diastolic Blood Pressure; HR, Heart Rate; TSH, Serum Thyroid-stimulating Hormone; FT4, Free T4; FT3, Free T3; TSTO, Testosterone; PRL, Prolactin; PRGE, Progesterone; LH, Luteinizing Hormone; E2, Estradiol; GH, Growth hormone; IGF-1, Insulin-like Growth factor-1; COR, cortisol; ACTH, adrenocorticotrophic hormone; PZC24, 24-hour urine free cortisol; IPSS, inferior petrosal sinus sampling; MRI, Magnetic Resonance Imaging. FT3 (range 3.5–6.5 pmol/L). FT4 (range 11.5–22.7 pmol/L). TSH (range 0.55–4.78 uIU/mL). TSTO (range female 0.5–2.6, male <50 years 4.94–32.01 nmol/L). FSH (range female 2.5–10.2, male 0.95–11.95 mIU/mL). PRL, (range female 59–619, male 72.66–407.4 uIU/mL). PRGE (range female 0.5–4.5, male 0.2–1.040 nmol/L). LH (range female, 1.9–12.5, male 0.57–12.07 mIU/mL). E2 (range female, 71.6–529.2, male 40.4–161.5 pmol/L). GH (range <8 ng/mL). IGF-1 (range 116–358 ng/mL). COR (8 Am range 118.6–618 nmol/L 0.4 Pm range 85.3–459.6 nmol/L). ACTH (8 Am range <10 pmol/L). PZC24 (range 153.2–789.4 nmol/ 24-h)—means the patient did not measured.
