# Supplementary material for: Development and Validation of a Deep Learning Algorithm to Automatic Detection of Pituitary Microadenoma From MRI
Source: Front Med (Lausanne). 2021 Nov 29;8:758690. doi: 10.3389/fmed.2021.758690 (PMC8666533; doi:10.3389/fmed.2021.758690)
Supplement: Supplementary Method — A detailed description of PM-CAD Model. PM-CAD, Pituitary microadenoma-computer-aided diagnosis. [file Data_Sheet_1.docx]

**Development and Validation a Deep Learning Algorithm to Automatic Detection of**

**Pituitary Microadenoma from Magnetic Resonance Imaging**

**Supplemental information**

## Supplementary information for Materials and Methods

### Pituitary Detection Model

Although pituitary occupies a small region of MR images, radiologists can diagnose microadenoma based on the appearance of such a small region. On the other hand, directly feeding the whole MR images into a deep learning based diagnosis method imposes a huge challenge since non-pituitary regions will hinder microadenoma diagnosis significantly. Therefore, we employ a detection model to locate the pituitary region before diagnosis. The design of the detection model potentially benefits the consequence task in two aspects:

- It enhances pituitary microadenoma (PM) feature by discarding irrelevant regions, and promotes the microadenoma diagnosis performance since the designed detection model can help the microadenoma diagnosis process focus on the pituitary region.
- It reduces the overfitting problem of our microadenoma diagnosis model with a limited amount of data.

**A.1. Architecture**

**
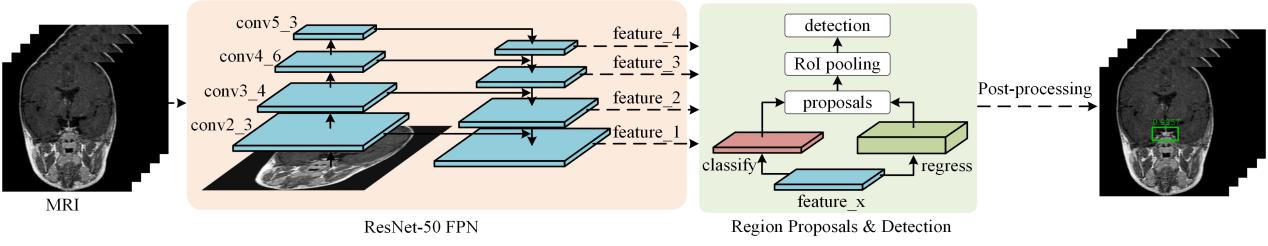
**

Figure 1. The pipeline of our pituitary detection model. The model consists of three parts: **ResNet-50 FPN** is used to extract the features in different scales, **Region Proposals & Detection** is used to generate the bounding boxes (which indicate pituitary regions), and **Post-processing** is used to refine the detection result.

Given consecutive MR images at the same anatomical section of the brain from the coronal dynamic enhancement T1-weighted imaging (T1WI) sequence of MRI scan, our pituitary detection model aims at locating the pituitary in each image. The pituitary detection model is built upon the Faster-RCNN ^[1]^ framework, and mainly consists of three parts: **ResNet-50 FPN** ^[2]^ extracts the multi-scale features from each image, **Region Proposals & Detection** produces the bounding box of pituitary from the multi-scale features, and **Post-processing** refines the detection results. (See Fig 1.)

**ResNet-50 FPN**

We use ResNet-50 FPN as our backbone to efficiently extract multi-scale features from each MR image. Specifically, ResNet-50 ^[3]^ is employed to process the input MR image, and four feature maps from conv2_3, conv3_4, conv4_6 and conv5_3 are further used as the input for the feature pyramid network (FPN). These four feature maps have 4×, 8×, 16×, 32× strided resolution compared to the input image.

FPN aggregates the above four feature maps, and produces four feature maps in different scales (i.e., feature_x where $x\in\{1,2,3,4\}$). The four feature maps are firstly processed by a 1×1 convolutional layer, to make them have the same number of channels. We denote the processed four feature maps as P_1, P_2, P_3 and P_4. The feature_4 is obtained by upsampling P4 with nearest interpolation. Feature_x (where $x\in\{2,3,4\}$) is obtained by upsampling the combination of the feature_(x-1) and the lateral feature map P_x. Therefore, four multi-scale features maps are used as the input for the following part.

**Region Proposals & Detection**

In this part, we process each feature map from ResNet-50 FPN independently, and the candidate bounding boxes indicating pituitary are produced. Specifically, given a feature map feature_x, two branch layers (each with one convolutional layer) are used to generate two feature maps. One branch is used to classify whether the anchor contains an object, and the another branch is used to regress the size (i.e., height, width) and position (i.e., x, y) from the default anchor. We obtain the bounding box proposals from these two feature maps using the non-maximum suppression (NMS) ^[1]^ algorithm to discard high-overlapping bounding boxes. Then, RoI pooling ^[1]^ is performed to aggregate the feature of feature_x within the bounding box proposals. The aggregated feature is further used to refine the classification and regression results, and the produced candidate bounding boxes of pituitary with confidence scores.

Each MR image has at most one pituitary. Therefore, from all candidate bounding boxes, we select the bounding box which has the highest confidence score. In order to reduce the false-positive rate of pituitary detection, we filter out the bounding box whose confidence score is smaller than a threshold $t$ (*t* = 0.5 in our experiments).

**Post-processing**

Among all MR images, the above procedure might fail to locate the pituitary in some images. To handle this problem, we refine the pituitary detection result by post-processing. Specifically, we average all the positions and sizes of the detected bounding boxes, which can be considered as a mean bounding box. Because the position and size of pituitary over different MR images do not change a lot, we assign the missed detected MR image with the mean bounding box.

**A.2. Experiment**

**Dataset**

We collect 666 MRI scan (performed by GE, Philips machines) from the Third Affiliated Hospital of Sun Yat-Sen University. 2718 MR images with pituitaries from the coronal dynamic enhancement T1WI sequence of MRI are selected. A neuroradiologist annotates each image with a bounding box indicating the pituitary region. We randomly split all MRI scan into a pituitary detection training set with 532 scans (2167 images) and a pituitary detection validation set with 134 scans (551 images).

As the evaluation metrics, we use AP@0.50 and AP@0.75 from the COCO Detection Benchmark ^[4]^. AP@0.50 and AP@0.75 stand for the Average Precision (AP) with intersection of union (IOU) thresholds of 0.5 and 0.75, respectively.

**Implementation**

The pituitary detection model is implemented with PyTorch, and trained on a computer with an NVIDIA TITAN RTX GPU. We use the SGD optimizer with a momentum of 0.9 for training. The initial learning rate is set to 0.005. The warm-up strategy is adapted, which linearly increases the learning rate from 0.001 to the initial value over each iteration at the first epoch. Then, the learning rate decreases by 0.1 per 3 epochs. The weight decay is set to 0.0005. We resize the input images into 256×256 and normalize into (0,1) based on the window level (WL) and window width (WW) set by neuroradiologist as preprocessing. Other configurations (e.g., loss function, default anchor setting) are the same as ^[1]^. We train the pituitary detection model for 20 epochs with a batch size of 16.

**Results**

Table 1 shows the pituitary detection performance on MRI achieved by our method. On the training set, our method achieves 0.9884 and 0.9078 in terms of AP@0.50 and AP@0.75, respectively. The performance of pituitary detection on the testing set is close to that of the training set. That is, our model achieves a good generalization capability on unseen data. Moreover, our detection model can accurately locate the pituitary region. When 0.5 is used as the IOU threshold to determine the success of pituitary detection, our method achieves an average precision of 97.83% over different levels of recall.

Table 1. Pituitary Detection Results on MRI

|  | AP@0.50 | AP@0.75 |
| --- | --- | --- |
| Training set | 0.9884 | 0.9078 |
| Testing set | 0.9783 | 0.8824 |

Figure 2 shows the prediction results of our pituitary detection model and the ground-truth labels of several MR images on the validation set. The results demonstrate our method can produce accurate prediction bounding boxes with high overlaps with the ground-truth.


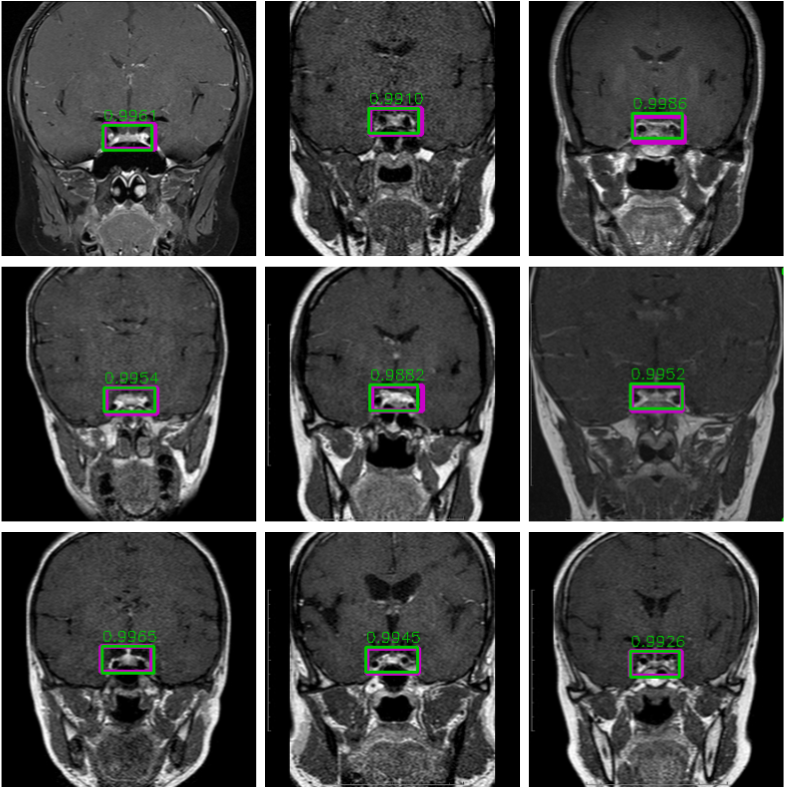


Figure 2. Some examples of pituitary detection on the validation set. Our method can produce accurate prediction bounding boxes (represented as green boxes) with high overlaps with the ground-truth (represented as purple boxes). Besides, the confidence scores produced by our pituitary detection model are also shown (i.e., the numbers near green boxes).

### Pituitary Microadenoma Diagnosis Model

Automatic diagnosis of PM with deep learning is highly challenging due to three main reasons: (1) **Low inter-class variance**. Microadenoma occupies a very small part of the pituitary, the difference between microadenoma and normal tissues mainly lies in fine-grained textures. Actually, normal pituitaries and pituitaries with microadenoma are very similar in most parts, it is therefore very hard to identify microadenoma even by radiologist. Consequently, the proposed model should be powerful enough to distinguish these minor differences. (2) **High intra-class variance**. PM vary significantly in terms of location, size, and shape. Consequently, the proposed model should be sufficiently representative to adapt to these variations. (3) **Limited training data**. Deep learning techniques usually require a large amount of training data to avoid overfitting. However, the insufficiency of PM MRI scan poses new challenges to the design of architecture and training skills of neural networks.

To handle these challenges, we propose a novel CNN (namely, PM-CAD) for PM diagnosis. In PM-CAD, we modify the ResNet architecture to preserve fine-grained features during forward propagation. Furthermore, an attention module is used to further improve the discriminativeness of feature representation. To handle the overfitting problem, histogram matching normalization, intensity shift data augmentation and label-smoothing loss are used.

**B.1. Architecture**

**
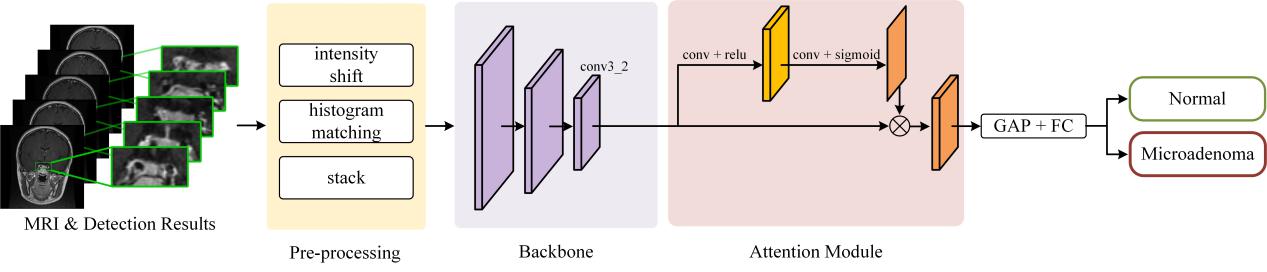
**

Figure 3. The pipeline of our microadenoma diagnosis model (PM-CAD). The model consists of three prats: **Pre-processing**, an improved **Backbone**, and an **Attention Module**. (conv: convolutional layer, relu: activation function, GAP: global average pooling, FC: fully connected layer.)

Given the consecutive MR images at the same anatomical position of the brain from the coronal dynamic enhancement T1WI sequence of MRI scan, our microadenoma diagnosis model aims at classifying them into normal or microadenoma. In particular, all MR images are processed using intensity shift as data augmentation and histogram matching normalization, then patches which only contain the pituitary are cropped from each MR image based on the detection results from the pituitary detection model. Then, all patches are resized and stacked as an image with multi-channels. Finally, our proposed CNN-based model processes these stacked pituitary patches and classifies them into normal or microadenoma.

In the following, we will first describe our CNN-based model with the improved **backbone** and an **attention module**, then highlight the intensity shift data augmentation and histogram matching normalization in **pre-processing**. Finally, we introduce the loss function used to train our CNN-based model.

**Backbone**

Most modern CNN models have two drawbacks when employing microadenoma diagnosis. (1) **Aggressive downsampling**. They use max-pooling layers or strided convolutions to downsample the feature maps. For example, the ResNet series use a large kernel convolution with a stride of 2 followed by a max-pooling layer at the beginning, and 4 strided convolutional layers in the following. This strategy can efficiently reduce the computational cost and save the memory. However, fine-grained features which are important in PM classification might be lost in aggressive downsampling. (2) **Large amount of trainable parameters**. Modern CNN models tend to contain a large amount of trainable parameters. Therefore, a large training dataset is required to achieve a sufficient performance. However, the limited data of microadenoma MRI scan impose the over-fitting problem. A lightweight model with fewer parameters might be more suitable for microadenoma diagnosis.

Therefore, we extend ResNet-18 from two aspects. First, we replace the large kernel convolution with 3×3 convolution at the beginning of the network. Second, we remove the max-pooling layer at the beginning and the last stages of convolution to preserve fine-grained features. Therefore, *conv4_2* feature map is obtained with a 8× strided resolution. Then a global average pooling (GAP) and a multi-layer perceptron (MLP) with softmax are used to produce the probability of PM. Experimental results show that this simple modification of ResNet-18 can efficiently improve the performance of PM diagnosis.

**Attention Module**

The vector at each position of *conv4_2* feature map is considered as the feature of a small patch from the input. Since the number of normal patches is larger than that of microadenoma, the global average pooling (GAP) which directly performs on this feature map might make the microadenoma feature overwhelming from the normal feature. Therefore, we introduce an attention module to augment the microadenoma feature before GAP.

Given a feature map $f$ of conv4_2, we employ a learnable convolution layer to produce a soft mask:

$$mask=sigmoid(conv_{2}(relu(conv_{1}(f))))$$

where $conv_{1}$ and $conv_{2}$ represent the 1×1 convolutional layers with 256 and 1 channels, respectively. This soft mask is further used to automatically select the prominent spatial areas of feature map $f$ by pixel-wise multiplication as follows:

$$f_{aug}=f\cdot mask$$

During training, this mask can learn to highlight the areas which have the discriminative features of microadenoma and suppress the irrelevant areas. Therefore, the augmented feature map contains the areas which is helpful for distinguishing the microadenoma and normal tissue.

The augmented feature map is further performed by GAP to aggregate spatial features. Then, the fully connected layers (FC) with softmax is used to produce the presence probability of microadenoma.

**Pre-processing**

Given the consecutive MR images with pituitary detection results, we first normalize them into (0,1) based on the window level (WL) and window width (WW). Then, data augmentation techniques such as Gaussian noise, translation, scaling, and the proposed intensity shift are performed during training. Furthermore, the proposed histogram matching normalization is used to align the histograms of all MR images. Finally, the patches only contain the pituitary are cropped from the MR images based on the detection result, and stacked as an image with mutli-channels. In the following, we will detail in our intensity shift data augmentation and histogram matching normalization.

**Intensity shift data augmentation (IS)**. To facilitate the domain knowledge from neuroradiologists, we normalize each MR image using WL and WW. This domain knowledge can help the network to focus on discriminative regions by assigning irrelevant pixels as background, and make the model converge faster. However, the normalization process decreases the generalization capacity on different hospitals. Different machines usually have different default values for WL and WW. Since WL and WW are adjusted by neuroradiologists based on their personal experience, the MR images normalized by different neuroradiologists from different machines in different hospitals have different distributions. Therefore, the model trained with the data from one hospital is difficult to generalize to other hospitals. To handle this limitation, we propose an intensity shift data augmentation approach to make the model insensitive to different WL and WW settings. In particular, we randomly shift the intensity of non-background pixels in MR images by adding a value within (0,0.1).

**Histogram matching normalization (HM)**. The MR images are acquired from the same anatomical section of brain. Therefore, the histograms between MR images are similar. In order to make the features between microadenoma and normal tissue more discriminative, we align the histograms using the histogram matching algorithm. Specifically, we select the histogram from one of the MR images as reference. Then, the histogram matching is performed to other MR image histograms to match the reference. In our experiments, we select the histogram from 3rd MR images among five consecutive MR images as the reference.

**Loss**

The cross-entropy loss is commonly used in classification. Specifically, the cross-entropy loss in binary classification can be formulated as:

$$loss_{ce}=-(1-y)\cdot\log(1-p)-y\cdot\log p$$

where $y\in\text{\{0，1\}}$ and $p\in(0,1)$ are the ground-truth label and the probability produced by the model. Usually, the probability is obtained by normalizing (e.g., softmax) the logits from the last layer of the model. The cross-entropy loss forces the logit to become an infinite value. That is, it pushes the distance of the learned feature between microadenoma and normal to be large, which potentially leads to over-fitting on the training data.

Motivated by ^[6]^, we use the label-smoothing loss as follows:

$$loss_{label-smoothing}=\left\{\begin{matrix} \begin{matrix} -\varepsilon\cdot\log(1-p)-(1-\varepsilon)\cdot\log p & y=1 \end{matrix} \\ \begin{matrix} \begin{matrix} -(1-\varepsilon)\log(1-p)-\varepsilon\cdot\log p & y=0 \end{matrix} & \end{matrix} \end{matrix}\right.$$

where $\varepsilon$ is a small number and we set $\varepsilon=0.1$ in our experiments. The label-smoothing loss can achieve the optimal performance when the logit is a finite value, this encourages the learned feature between microadenoma and normal more compact.

**B.2. Application on MRI Scan**

In this subsection, we will describe the approach for adapting our method to MRI scan.

Usually, a coronal dynamic enhancement T1WI sequence of MRI scan (DICOM) is used to diagnose PM. These images in a sequence can be divided into groups, and different groups focus on different anatomical sections of the pituitary. 2 neuroradiologistes and 1 neurosurgeon examine all MR images of each pituitary section, and diagnoses whether microadenomas present in this section. The MRI scan is considered as normal if the microadenoma cannot be found in any pituitary sections.

Following the same idea, we adapt our PM-CAD to the MR images as to achieve end-to-end diagnosing without any human intervention. Specifically, the proposed method first inspects each section of the pituitary gland based on the learned feature, and then performs ‘OR’ operation based on the classification result of all sections. Similarly, the MR image is considered normal if and only if all sections of the pituitary are diagnosed as normal by our PM-CAD.

**B.3. Experiment**

**Dataset**

1,520 participants with MRI scan were selected. The coronal dynamic enhancement T1WI sequence of MRI scan were used to diagnose PM in our experiments. All participants were split into 5 parts, that is:

The Development Dataset: It is used to train the deep learning models.

The Testing Dataset: It is used to tune the hyper-parameters (e.g., learning rate, number of training epochs) of the models and select the best model during training.

Validation A: It is used to evaluate the generalization ability of our proposed PM-CAD system on MRI scan from different hospitals.

Validation B: It is used to compare the performances between radiologists and our PM-CAD system in PM diagnosis.

Validation C: It is used to test the diagnosis performances of our PM-CAD system on radilologists misdiagnosed cases.

**Evaluation metric**

To compare the performance of PM diagnosis between different models, we use the area under receiver operating characteristic curve (AUC) score (which is independent of probability threshold). In addition, F1-score, accuracy, sensitivity, specificity, positive predictive value (PPV), negative predictive value (NPV), error, positive likelihood ratio (PLR) and negative likelihood ratio (NLR) are used as metrics for performane evaluation.

The 95% confidence interval (CI) is also calculated for each metric. In particular, CI for AUC is calculated using bootstrap confidence intervals ^[10]^ (resample 50,000 times with replacement). CIs for F1-score, accuracy, sensitivity, specificity, PPV and NPV are calculated using the Clopper-Pearson interval ^[11]^ because it is common for calculating binomial confidence intervals. CIs for PLR and NLR are calculated using the “Log method” ^[7]^.

**Implementation**

We implement the microadenomas diagnosis model with PyTorch, and train our PM-CAD on NVIDIA TITAN RTX GPUs. We use the SGD optimizer with a momentum of 0.9. The initial learning rate is set to 0.02 and decreases by 0.99 per epoch. The weight decay is set to 0.0005. We train our PM-CAD for 500 epochs with a batch size of 16. We set the MLP in backbone to have two layers with 256 and 1 channels, respectively.  The $\varepsilon$ in our label-smoothing loss is set to 0.1. We fine-tune our PM-CAD model from the weights pre-trained on ImageNet ^[8]^.

The probability threshold of the model is selected based on the Youden Index ^[5]^ of the Receiver Operating Curve (ROC). Specifically, given the probabilities produced by the model and their corresponding ground-truth labels, we can first calculate the True Positive Rates (TPR) and the False Positive Rates (FPR) under different levels of threshold t. Then, the Youden Index at threshold t is defined as:

$$YoudenIndex_{t}=TPR_{t}-FPR_{t}$$

Finally, the threshold producing the largest Youden Index is considered as the optimal probability threshold $\hat{t}$:

$$\hat{t}=\begin{matrix} \mathop{\mathrm{argmax}}\limits_{t} & YoudenIndex_{t} \end{matrix}$$

**Ablation Study**

We provide the ablation study on the intensity shift data augmentation and the histogram matching normalization. We train our PM-CAD with or without intensity shift data augmentation and histogram matching normalization using training dataset, and evaluate it on Validation C. Table 2 shows the accuracy has improved by 2% in hospital 3 and hospital 2, when equipped with HM and IS. And HM and IS do not improve the performance on the data from hospital 1. That is because, the training dataset and test data from hospital 1 have the same source which is the Third Affiliated Hospital of Sun Yat-Sen University, resulting in consistent normalization set by WL and WW. The result shows that the proposed HM and IS can eliminate the effect of inconsistent normalization from different hospitals. With HM and IS, our PM-CAD demonstrate a good generalization capacity, achieving above 92% accuracy of different hospitals.

Table 2. Accuracy of our PM-CAD (w/ or w/o HM, IS) on Validation A (from 3 different hospitals).

|  | hospital 1 | hospital 2 | hospital 3 |
| --- | --- | --- | --- |
| w/o HM, IS | 138/143 | 141/155 | 131/144 |
| w/ HM, IS | 138/143 | 143/155 | 133/144 |

**References**

1. Ren S, He K, Girshick R, Sun J. Faster R-CNN: Towards Real-Time Object Detection with Region Proposal Networks. IEEE Trans Pattern Anal Mach Intell. 2017 Jun; 39(6):1137-1149. PMID: **27295650.**

2. Lin TY, Dollar Piotr, Girshick Ross, et al. Feature Pyramid Networks for Object Detection. Proceedings of the IEEE Conference on Computer Vision and Pattern Recognition (CVPR). 2017, pp. 2117-2125.
3. He K, Zhang X, Ren S, et al. Deep Residual Learning for Image Recognition. Proceedings of the IEEE Conference on Computer Vision and Pattern Recognition (CVPR).2016, pp. 770-778.

4. Lin TY, Maire M, Belongie S, et al. Microsoft COCO: Common Objects in Context. Computer Vision-ECCV. 2014, pp. 740-755.

5. Youden WJ. Index for rating diagnostic tests. Cancer. 1950 Jan;3(1):32-5.

6. Szegedy C, Vanhoucke V, Loffe S, et al. Rethinking the Inception Architecture for Computer Vision. Proceedings of the IEEE Conference on Computer Vision and Pattern Recognition (CVPR). 2016, pp. 2818-2826.

7. Altman D, Machin D, Bryant T, et al. Statistics with confidence. BMJ Books. 2000, 2nd ed.

8. Russakovsky O, Deng J, Su H, et al. ImageNet Large Scale Visual Recognition Challenge. International Journal of Computer Vision. 2015, 115, 211-252.

9. Zhou Z, Sodha V, Siddiquee MMR, et al. Models Genesis: Generic Autodidactic Models for 3D Medical Image Analysis. [Medical Image Computing and Computer Assisted Intervention.](https://link.springer.com/book/10.1007/978-3-030-32251-9) 2019, pp. 384-393.

10. Thomas J. Bootstrap confidence intervals. Statistical Science. 1996, 11, 189-212.

11. Puza B, O'Neill T. Generalised Clopper–Pearson confidence intervals for the binomial proportion. Journal of Statal Computation & Simulation. 2006, 76, 489-508.

**Legend for Supplementary Figures**

**Supplementary Figure 1. Cases of 4 misdiagnosed pituitary microadenomas.** (A) 4 consecutive pituitary MRI scans over a period of 20 months in a misdiagnosed patient with pituitary microadenoma. The radiologists have not detected the pituitary microadenoma during the first 3 MRI examinations. A functional microadenoma has been localized by the subsequent ACTH examination of the inferior petrosal sinus in the region of right pituitary gland. On the 4th MRI scanning, two microadenoma are detected by radiologist. (B) Additional 3 cases of misdiagnosed microadenoma. Patient 1 has a very small microadenoma with a diameter < 3 mm. Patient 2 has an irregularly shaped microadenoma. Patient 3 has two microadenoma (with diameters of 2.8 mm and 6.1 mm, respectively) and the smaller one was misdiagnosed. The comprehensive clinical data for patients were listed in Supplementary Table 1. ACTH: Adrenocorticotropic Hormone. MRI: magnetic resonance imaging. T1WI-COR: T1 weighted imaging-coronal. MRI bar = 5 mm. The yellow arrow and the area inside the red circle represent adenomas.

**Supplementary Figure 2. Workflow diagram for the validation datasets.** PM: pituitary microadenoma. MRI: magnetic resonance imaging.

**Supplementary Figure 3. Overview of our PM-CAD system.** (A) First the MR images are fed into our PM-CAD system for automatic diagnosis. The proposed PM-CAD system consists of two models: (B) the pituitary detection model localizes the pituitary region in cerebral MRI. The MR images are processed with multiple convolutional layers and two maps (classification map is used to predict the center and the regression map is used to refine the height and width of the rectangle box) are produced to predict a rectangle box enclosing the pituitary region. The pituitary rectangle region is cropped, stacked, and then fed into the PM diagnosis model. (C) It employs the proposed PM-CAD model to extract features. A softmax layer is employed to transform the feature into the presence probability of PM. CAD: computer-aided diagnosis. MRI: magnetic resonance imaging. MR: magnetic resonance. PM: pituitary microadenoma.

**Supplementary Figure 4. Performance of the PM-CAD system on the training datasets.** (A). Accuracy curves achieved by the PM-CAD system on the development and testing datasets. (B). Cross entropy loss curves achieved by the PM-CAD system on the development and testing datasets. We train the PM-CAD system for 500 epochs.

**Supplementary Figure 5. The PM-CAD system outperforms 6 radiologists in AUC of PM diagnosis.** (A) ROC and AUC: ROC curve shows the true positive rates (sensitivity) with respect to different false-positive rates (1-specificity). The ROC curve shows that the PM-CAD system outperforms 6 radiologists. The AUC of PM-CAD system is 95.6% better than our best radiologist#6 (AUC 95.0%). (B) Weighted error. A penalty weight of 2 is applied to false-negatives and a penalty weight of 1 is assigned to false-positives. The PM-CAD system produces a weighted error of 10%, whereas the radiologists produce a weighted error of 21.67%. (C & D) The negative likelihood ratio and the positive likelihood ratio: The negative likelihood ratio is defined as the false-negative rate over the true negative rate, so that a decreasing likelihood ratio < 1 indicated increasing probability the absence of PM. The positive likelihood ratio is defined as the true positive rate over the false-positive rate, so that an increasing likelihood ratio > 1 indicated increasing probability the diagnosis of PM. The confidence intervals show that the PM-CAD system demonstrates statistically better screening performance in terms of both negative likelihood ratio and positive likelihood ratio than radiologists. Radiologist 1 & 2: with < 5 years professional experience, Radiologist 3 & 4: with 5 - 10 years professional experience, Radiologist 5 & 6: with > 10 years professional experience. PM, pituitary microadenoma; receiver operating characteristics (ROC); the area under ROC curve (AUC).

**Supplementary Figure 6. The MRI and histological validation of double positive and false-negative cases.** (A, B) 3 double positive and 3 false-negative cases, which were functional PM, as confirmed by subsequent pathological examination. The comprehensive clinical data for these patients are listed in Supplementary Table 5. PM: pituitary microadenoma. MRI: magnetic resonance imaging. AI: Artificial intelligence. HE: hematoxylin and eosin. ACTH: adrenocorticotropic hormone. GH: growth hormone. TSH: thyroid stimulating hormone. PRL: prolactin. MR bar = 5mm. Pathology bar =100 μm. The yellow arrow indicates a pituitary microadenoma.

**Supplementary Figure 7. The browser-based software to aide the diagnosis of PM.** As long as we upload the pituitary MR images (DICOM), the software will tell you whether the patient suffering from PM disease. This browser based tool can be accessed at <http://82.157.181.77/> .

| Supplementary Table 1. The workload of radiologists with different professional experience in human-computer competition. | | | | | | | | |
| --- | --- | --- | --- | --- | --- | --- | --- | --- |
| Years of clinical work | Professional Experience | | | | | | | |
|  | < 5 years | |  | 5 to 10 years | |  | > 10 years | |
|  | Radiologist1 | Radiologist2 |  | Radiologist3 | Radiologist4 |  | Radiologist5 | Radiologist6 |
| The reading numbers of cranial MR and CT images every month | 885±93 | 861±87 |  | 916±85 | 943±96 |  | 949±97 | 963±104 |
| The reading numbers of pituitary MR images every month | 95±11 | 102±15 |  | 113±18 | 135±27 |  | 138±22 | 157±39 |
| Note: All participating radiologists are general radiologists (no specialisation). Workload analysis was performed on the participating radiologists for one year. | | | | | | | | |
|  |  |  |  |  |  |  |  |  |

| Supplementary Table 2. Confusion Matrices for testing and validation of dataset A (internal and external dataset). | | | | | | | | | | | | |
| --- | --- | --- | --- | --- | --- | --- | --- | --- | --- | --- | --- | --- |
|  | Truth | Testing set | |  | Temporal internal validation | |  | Geographical external validation | | | | |
|  |  | Adenoma | Normal |  | Set A1, Hospital 1 | |  | Set A2, Hospital 2 | |  | Set A3, Hospital 3 | |
|  |  |  |  |  | Adenoma | Normal |  | Adenoma | Normal |  | Adenoma | Normal |
| Prediction | Adenoma | 64 (a) | 9 (b) |  | 48 | 2 |  | 55 | 9 |  | 51 | 8 |
|  | Normal | 2 (c) | 120 (d) |  | 3 | 90 |  | 3 | 88 |  | 3 | 82 |
| Note: Data are numbers of images. a, true-positive; b, false-positive; c, false-negative; d, true-negative. | | | | | | | | | | | | |

| Supplementary Table 3. The diagnostic performance for Human-computer competition according to temporal validation set B (n=100). | | | | | | | | |
| --- | --- | --- | --- | --- | --- | --- | --- | --- |
| Evaluation Metrics |  | PM-CAD | Radiologist 1 | Radiologist 2 | Radiologist 3 | Radiologist 4 | Radiologist 5 | Radiologist 6 |
| F1 score |  | 0.9388 (92/98) | 0.7551 (74/98) | 0.7879 (78/99) | 0.8571 (84/98) | 0.8824 (90/102) | 0.9091 (90/99) | 0.9495(94/99) |
|  |  | [0.8715~0.9772] | [0.6579~0.8364] | [0.6942~0.8636] | [0.7719~0.9196] | [0.8035~0.9377] | [0.8344~0.9576] | [0.8861~0.9834] |
|  |  |  |  |  |  |  |  |  |
| Accuracy |  | 0.9400 (94/100) | 0.7600 (76/100) | 0.7900 (79/100) | 0.8600 (86/100) | 0.8800 (88/100) | 0.9100 (91/100) | 0.9500(95/100) |
|  |  | [0.8740~0.9777] | [0.6643~0.8398] | [0.6971~0.8651] | [0.7763~0.9213] | [0.7998~0.9364] | [0.8360~0.9580] | [0.8872~0.9836] |
|  |  |  |  |  |  |  |  |  |
| Sensitivity |  | 0.9200 (46/50) | 0.7400 (37/50) | 0.7800 (39/50) | 0.8400 (42/50) | 0.9000 (45/50) | 0.9000 (45/50) | 0.9400(47/50) |
|  |  | [0.8077~0.9778] | [0.5965~0.8537] | [0.6400~0.8847] | [0.7089~0.9283] | [0.7819~0.9667] | [0.7819~0.9667] | [0.8345~0.9874] |
|  |  |  |  |  |  |  |  |  |
| PPV |  | 0.9583 (46/48) | 0.7708 (37/48) | 0.7959 (39/49) | 0.8750 (42/48) | 0.8654 (45/52) | 0.9184 (45/49) | 0.9592(47/49) |
|  |  | [0.8575~0.9949] | [0.6269~0.8797] | [0.6566~0.8976] | [0.7475~0.9527] | [0.7421~0.9441] | [0.8040~0.9773] | [0.8602~0.9950] |
|  |  |  |  |  |  |  |  |  |
| Specificity |  | 0.9600 (48/50) | 0.7800 (39/50) | 0.8000 (40/50) | 0.8800 (44/50) | 0.8600 (43/50) | 0.9200 (46/50) | 0.9600(48/50) |
|  |  | [0.8629~0.9951] | [0.6404~0.8847] | [0.6628~0.8997] | [0.7569~0.9547] | [0.7326~0.9418] | [0.8077~0.9778] | [0.8629~0.9951] |
|  |  |  |  |  |  |  |  |  |
| NPV |  | 0.9231 (48/52) | 0.7500 (39/52) | 0.7843 (40/51) | 0.8462 (44/52) | 0.8958 (43/48) | 0.9020 (46/51) | 0.9412(48/51) |
|  |  | [0.8146~0.9786] | [0.6105~0.8597] | [0.6468~0.8871] | [0.7192~0.9312] | [0.7734~0.9653] | [0.7859~0.9674] | [0.8376~0.9877] |
| Note: Unless otherwise specified, data are percentages, with numbers of images in parentheses and 95% confidence intervals in brackets. F1 score, the harmonic mean of PPV and sensitivity. NPV, negative predictive value. PPV, positive predictive value. Radiologist 1 & 2, < 5 years professional experience; Radiologist 3 & 4, 5 - 10 years professional experience; Radiologist 5 & 6, > 10 years professional experience. | | | | | | | | |
|  |  |  |  |  |  |  |  |  |

| Supplementary Table 4. Confusion Matrices for Human-computer competition according to temporal validation set B (n=100) | | | | | | | | | | | | | | | |  |  |  |  |  |  |
| --- | --- | --- | --- | --- | --- | --- | --- | --- | --- | --- | --- | --- | --- | --- | --- | --- | --- | --- | --- | --- | --- |
|  | Truth | PM-CAD | |  | Radiologist 1 | |  | Radiologist 2 | |  | Radiologist 3 | |  | Radiologist 4 | |  | Radiologist 5 | |  | Radiologist 6 | |
|  |  | Adenoma | Normal |  | Adenoma | Normal |  | Adenoma | Normal |  | Adenoma | Normal |  | Adenoma | Normal |  | Adenoma | Normal |  | Adenoma | Normal |
| Prediction | Adenoma | 46 (a) | 2 (b) |  | 37 | 11 |  | 39 | 10 |  | 42 | 6 |  | 45 | 7 |  | 45 | 4 |  | 47 | 2 |
|  | Normal | 4 (c) | 48 (d) |  | 13 | 39 |  | 11 | 40 |  | 8 | 44 |  | 5 | 43 |  | 5 | 46 |  | 3 | 48 |
| Note: Data are numbers of images. a, true-positive; b, false-positive; c, false-negative; d, true-negative. | | | | | | | | | | | | |  |  |  |  |  |  |  |  |  |

| Supplementary Table 5. The patient clinical data in SFigure 1 and SFigure 6 | | | | | | | | | | | | | | |
| --- | --- | --- | --- | --- | --- | --- | --- | --- | --- | --- | --- | --- | --- | --- |
| Characteristics | SFig 1a | SFig 1b, Patient 1 | SFig 1b, Patient 2 | SFig 1b, Patient 3 | | SFig 6, Patient 1 | | SFig 6, Patient 2 | | SFig 6, Patient 3 | | SFig 6, Patient 4 | SFig 6, Patient 5 | SFig 6, Patient 6 |
| Diagnosis | Cushing's disease | PM | PM | PM | | Cushing's disease | | Acromegaly | | Prolactinoma | | Cushing's disease | Cushing's disease | Pituitary TSH adenoma |
| Gender | Female | Female | Male | Female | | Female | | Female | | Female | | Female | Female | Male |
| Age, years | 45 | 35 | 39 | 43 | | 30 | | 37 | | 29 | | 37 | 29 | 45 |
| Clinical feature | Infertility, diabetes, menstrual irregularity | No special clinical feature | Headache | No special clinical feature | | Diabetes, hypertension, menstrual irregularity | | Face changes, fingers and toes become coarsen | | Headache, menstrual irregularity, galactorrhea | | Weight increase, menstrual disorder | Menstrual disorder, polyphagia | Dizzy, palpitation, sweating, irritability |
| Clinical signs | Truncal obesity, thin skin, striae, ecchymosis | No special signs | No special signs | No special signs | | Truncal obesity, acne, thin skin, striae | | Face broad, nose thickened, fingers stubby | | No diplopia or visual field defect | | Truncal obesity, facial acne, abdominal purple striae | Truncal obesity, full moon face, buffalo back, lap striae | Heart rate 100 beats per minute, degree I goiter |
| BMI, kg/m^2^ | 19.60 | 20.83 | 24.60 | 25.81 | | 23.45 | | 22.64 | | 25.85 | | 23.23 | 22.43 | 20.74 |
| Baseline SBP, mmHg | 168 | 112 | 135 | 133 | | 150 | | 106 | | 134 | | 124 | 106 | 124 |
| Baseline DBP, mmHg | 109 | 76 | 93 | 97 | | 100 | | 71 | | 79 | | 78 | 76 | 82 |
| **Blood biochemical indices** | | | | | | | | | | | | | | |
| HbA1c% | 14.20 | 5.50 | 5.30 | | 4.90 | | 9.30 | | 5.50 | | 5.80 | 5.30 | 5.50 | 4.80 |
| FT3, pmol/L. | 3.75 | 4.32 | 4.66 | | 5.92 | | 2.28 | | 4.64 | | 4.65 | 3.77 | 3.53 | 10.23 |
| FT4, pmol/L. | 19.60 | 20.34 | 13.56 | | 15.32 | | 13.14 | | 20.28 | | 12.20 | 16.34 | 14.32 | 35.26 |
| TSH, uIU/mL | 5.31 | 2.12 | 3.77 | | 1.93 | | 0.31 | | 0.17 | | 2.26 | 0.67 | 0.74 | 11.42 |
| TSTO, nmol/L | 2.82 | 1.84 | 17.60 | | 1.23 | | 0.99 | | 1.22 | | 1.90 | 2.78 | 1.61 | 7.47 |
| FSH, mIU/mL | 3.49 | 3.98 | 3.20 | | 4.92 | | 2.36 | | 1.20 | | 5.77 | 3.93 | 2.52 | 2.97 |
| PRL, uIU/mL | 93.82 | 329.47 | 197.87 | | 274.65 | | 124.87 | | 163.25 | | ﹥4240 | 186.83 | 248.04 | 315.78 |
| PRGE, nmol/L | 2.94 | 3.28 | <0.10 | | 1.93 | | 3.79 | | 2.07 | | 1.01 | 2.02 | 6.06 | 0.60 |
| LH, mIU/mL | 5.27 | 6.32 | 2.91 | | 3.74 | | 1.48 | | 0.14 | | 7.27 | 5.55 | 0.60 | 1.68 |
| E2, pmol/L | 451.65 | 234.75 | 73.00 | | 156.32 | | 328.48 | | 189.93 | | 616.00 | 192.88 | 226.60 | 86.00 |
| GH, ng/mL | - | 0.25 | 0.07 | | - | | 0.71 | | 25.00 | | <0.05 | - | - | 1.99 |
| IGF-1, ng/mL | - | - | 121.00 | | - | | - | | 802.00 | | 144.00 | - | - | 160.00 |
| COR,8Am, nmol/L | 1500.74 | 356.98 | 418.95 | | 467.35 | | 939.71 | | 334.76 | | 800.00 | 757.99 | 844.25 | 337.63 |
| COR,4Pm, nmol/L | 919.63 | 176.23 | 199.53 | | 234.19 | | 666.60 | | 369.51 | | 262.19 | 737.58 | 769.63 | 117.71 |
| COR,0Am, nmol/L | 918.02 | 75.41 | 47.18 | | 62.56 | | 424.71 | | 230.00 | | 29.96 | 661.26 | 715.14 | - |
| ACTH,8Am, pmol/L | 21.80 | 3.96 | 2.93 | | 4.67 | | 25.80 | | 3.11 | | 6.99 | 10.30 | 11.80 | 4.48 |
| PZC24, nmol/ 24-hour | 19607.00 | - | - | | - | | 2744.32 | | - | | - | 2507.02 | 1977.92 | 449.68 |
| **1-mg dexamethasone suppression test** | | | | | | | | | | | | | | |
| COR,8Am, nmol/L | 1386.93 | - | - | | - | | 812.98 | | - | | - | 573.04 | 692.44 | - |
| **8-mg dexamethasone suppression test** | | | | | | | | |  | |  |  |  |  |
| PZC24, nmol/ 24-hour | 3179.65 | - | - | | - | | 99.96 | | - | | - | 224.67 | 141.04 | - |
| MRI scans | PM | PM | PM | | PM | | PM | | PM | | PM | PM | PM | PM |
| ACTH, IPSS | ﹥278.00 | - | - | | - | | - | | - | | - | - | 130.40 | - |
| Transsphenoidal resection | Yes | No | No | | No | | Yes | | Yes | | Yes | Yes | Yes | Yes |
| Immunohistochemistry | ACTH (+) | - | - | | - | | ACTH (+) | | GH (+) | | PRL (+) | ACTH (+) | ACTH (+) | TSH (+) |
| **Postoperation blood biochemical indices** | | | | | | | | | | | | | | |
| TSH | 1.70 | - | - | | - | | 2.10 | | 2.40 | | 1.30 | 3.20 | 1.80 | 2.50 |
| PRL | 234.56 | - | - | | - | | 172.48 | | 198.27 | | 212.47 | 258.23 | 317.48 | 102.39 |
| GH | - | - | - | | - | | - | | 2.42 | | - | - | - | - |
| IGF-1 | - | - | - | | - | | - | | 247.00 | | - | - | - | - |
| ACTH | 6.20 | - | - | | - | | 7.30 | | 2.39 | | 4.82 | 2.53 | 1.69 | 9.23 |
| COR, 8Am | 313.41 | - | - | | - | | 219.45 | | 349.32 | | 298.17 | 122.98 | 296.92 | 389.18 |
| Note: PM, pituitary microadenoma. BMI, Body Mass Index. SBP, Systolic Blood Pressure. DBP, Diastolic Blood Pressure. HR, Heart Rate. TSH, Serum Thyroid-stimulating Hormone. FT4, Free T4. FT3, Free T3. TSTO, Testosterone. PRL, Prolactin. PRGE, Progesterone. LH, Luteinizing Hormone. E2, Estradiol. GH, Growth hormone. IGF-1, Insulin-like Growth factor-1. COR, cortisol. ACTH, adrenocorticotrophic hormone. PZC24, 24-hour urine free cortisol. IPSS, inferior petrosal sinus sampling. MRI, Magnetic Resonance Imaging. FT3 (range 3.5-6.5 pmol/L). FT4 (range 11.5-22.7 pmol/L). TSH (range 0.55-4.78 uIU/mL). TSTO (range female 0.5-2.6, male <50year 4.94-32.01 nmol/L). FSH (range female 2.5- 10.2, male 0.95-11.95 mIU/mL). PRL, (range female 59- 619, male 72.66-407.4 uIU/mL). PRGE (range female 0.5-4.5, male 0.2-1.040 nmol/L). LH (range female, 1.9- 12.5, male 0.57-12.07 mIU/mL). E2 (range female, 71.6- 529.2, male 40.4-161.5 pmol/L). GH (range <8 ng/mL). IGF-1 (range 116-358 ng/mL). COR (8Am range 118.6- 618 nmol/L.4Pm range 85.3- 459.6 nmol/L). ACTH (8Am range <10 pmol/L). PZC24 (range 153.2-789.4 nmol/ 24-hour.) - means the patient did not measured. | | | | | | | | | | | | | | |
