# Supplementary figures and images for: Development and Validation of a Deep Learning Algorithm to Automatic Detection of Pituitary Microadenoma From MRI
Source: Front Med (Lausanne). 2021 Nov 29;8:758690. doi: 10.3389/fmed.2021.758690 (PMC8666533; doi:10.3389/fmed.2021.758690)

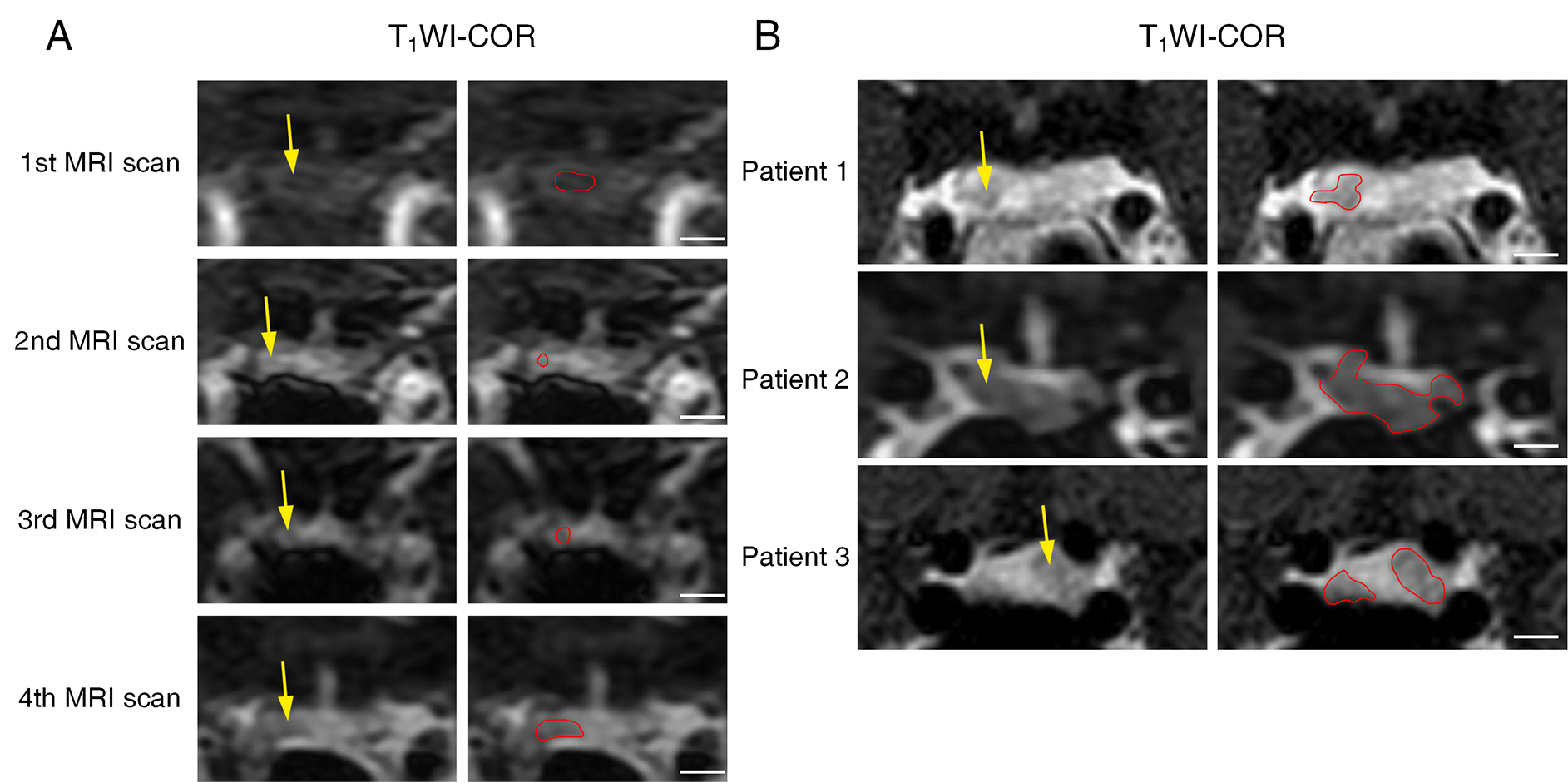

Supplement: Supplementary Figure 1 — Cases of 4 misdiagnosed pituitary microadenomas. (A) 4 consecutive pituitary MRI scans over a period of 20 months in a misdiagnosed patient with pituitary microadenoma. The radiologists have not detected the pituitary microadenoma during the first 3 MRI examinations. A functional microadenoma has been localized by the subsequent ACTH examination of the inferior petrosal sinus in the region of right pituitary gland. On the 4th MRI scanning, two microadenoma are detected by radiologist. (B) Additional 3 cases of misdiagnosed microadenoma. Patient 1 has a very small microadenoma with a diameter <3 mm. Patient 2 has an irregularly shaped microadenoma. Patient 3 has two microadenoma (with diameters of 2.8 mm and 6.1 mm, respectively) and the smaller one was misdiagnosed. The comprehensive clinical data for patients were listed in Supplementary Table 1. ACTH, Adrenocorticotropic Hormone; MRI, magnetic resonance imaging; T1WI-COR, T1 weighted imaging-coronal. MRI bar = 5 mm. The yellow arrow and the area inside the red circle represent adenomas. [file Image_1.TIF]

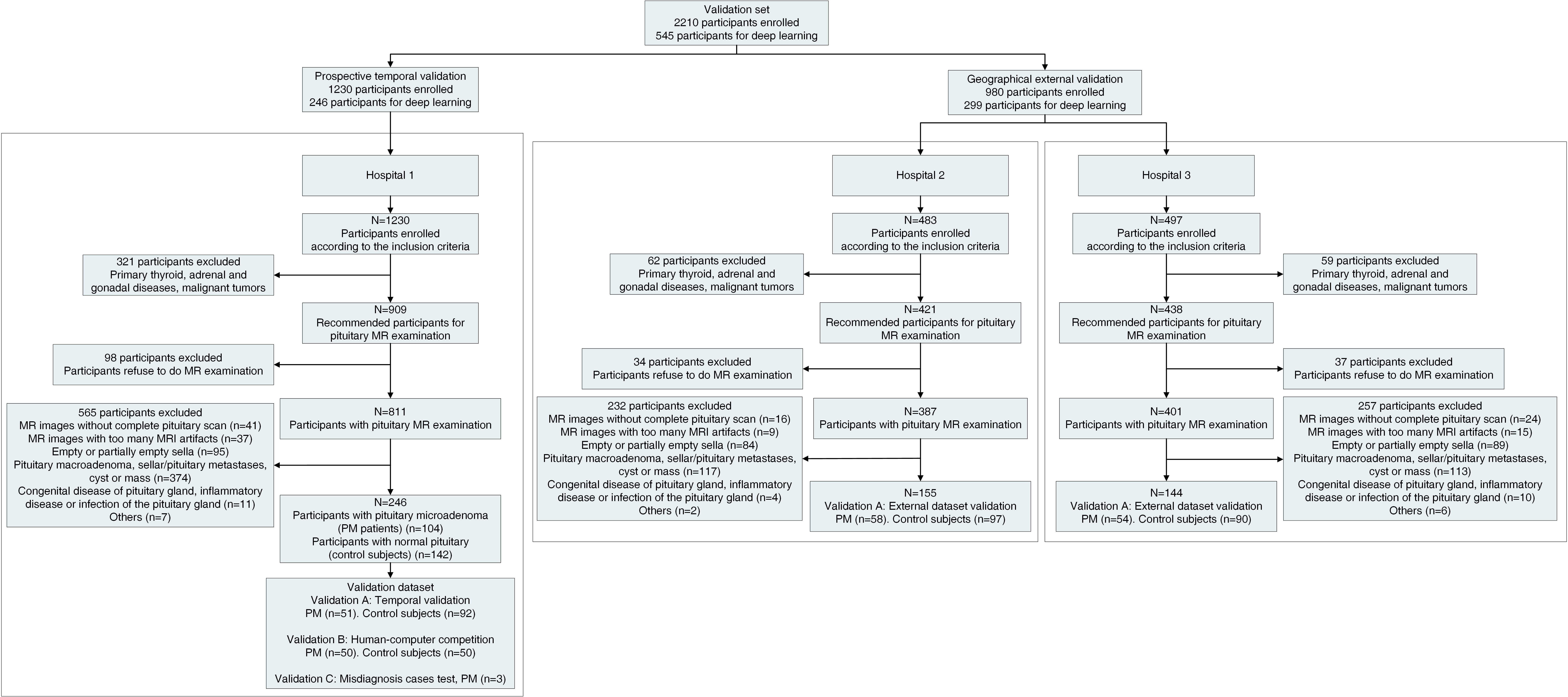

Supplement: Supplementary Figure 2 — Workflow diagram for the validation datasets. PM, pituitary microadenoma; MRI, magnetic resonance imaging. [file Image_2.TIF]

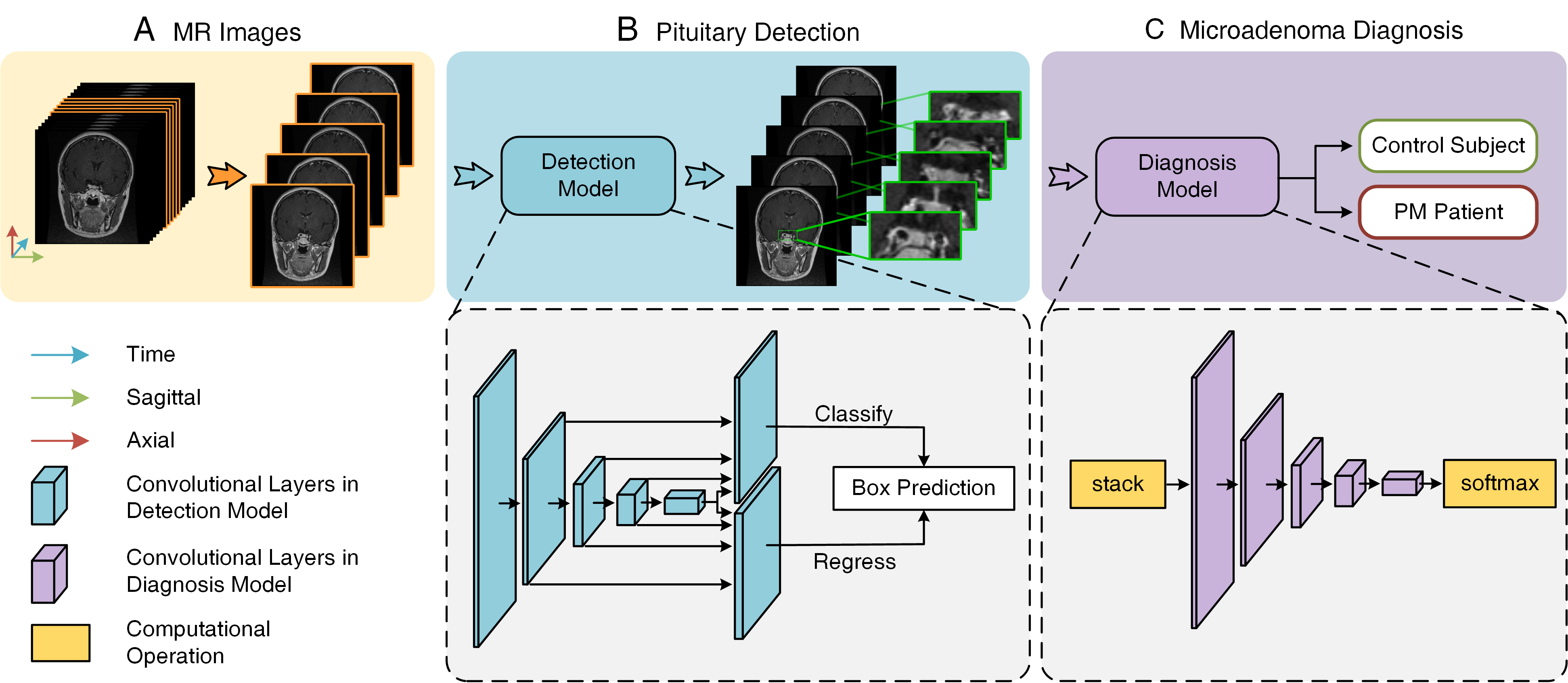

Supplement: Supplementary Figure 3 — Overview of our PM-CAD system. (A) First the MR images are fed into our PM-CAD system for automatic diagnosis. The proposed PM-CAD system consists of two models: (B) the pituitary detection model localizes the pituitary region in cerebral MRI. The MR images are processed with multiple convolutional layers and two maps (classification map is used to predict the center and the regression map is used to refine the height and width of the rectangle box) are produced to predict a rectangle box enclosing the pituitary region. The pituitary rectangle region is cropped, stacked, and then fed into the PM diagnosis model. (C) It employs the proposed PM-CAD model to extract features. A softmax layer is employed to transform the feature into the presence probability of PM. CAD, computer-aided diagnosis; MRI, magnetic resonance imaging; MR, magnetic resonance; PM, pituitary microadenoma. [file Image_3.TIF]

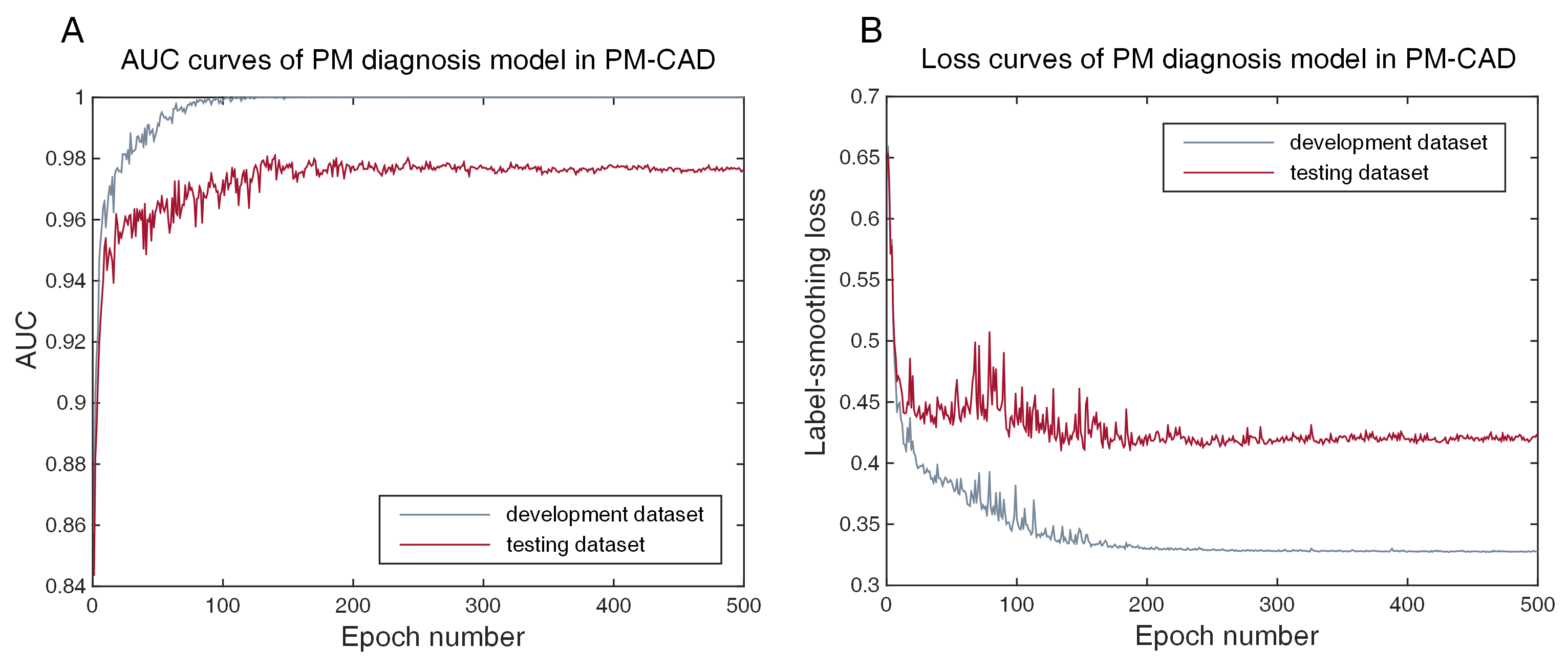

Supplement: Supplementary Figure 4 — Performance of the PM-CAD system on the training datasets. (A) Accuracy curves achieved by the PM-CAD system on the development and testing datasets. (B) Cross entropy loss curves achieved by the PM-CAD system on the development and testing datasets. We train the PM-CAD system for 500 epochs. [file Image_4.TIF]

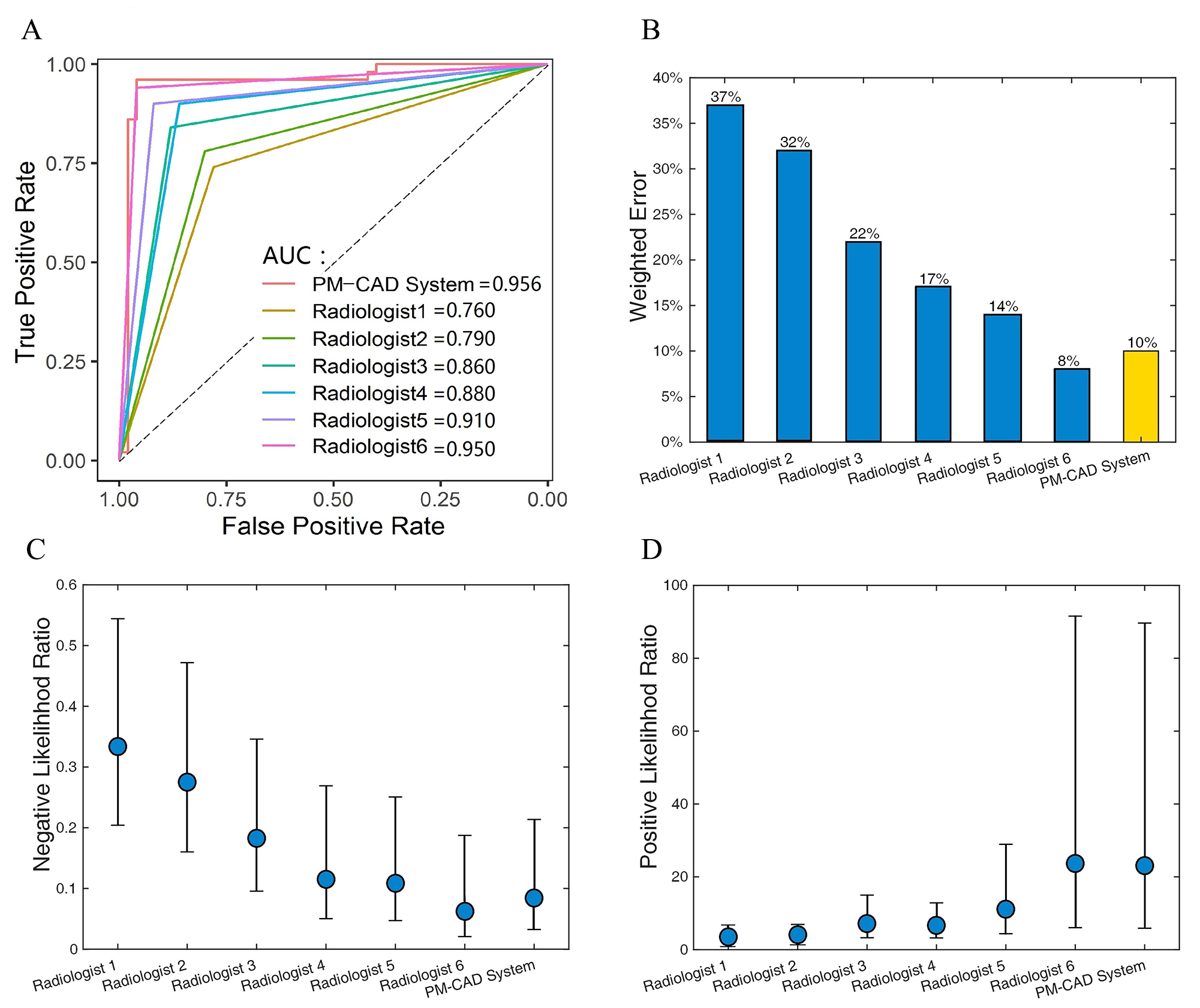

Supplement: Supplementary Figure 5 — The PM-CAD system outperforms 6 radiologists in AUC of PM diagnosis. (A) ROC and AUC: ROC curve shows the true positive rates (sensitivity) with respect to different false-positive rates (1-specificity). The ROC curve shows that the PM-CAD system outperforms 6 radiologists. The AUC of PM-CAD system is 95.6% better than our best radiologist#6 (AUC 95.0%). (B) Weighted error. A penalty weight of 2 is applied to false-negatives and a penalty weight of 1 is assigned to false-positives. The PM-CAD system produces a weighted error of 10%, whereas the radiologists produce a weighted error of 21.67%. (C,D) The negative likelihood ratio and the positive likelihood ratio: The negative likelihood ratio is defined as the false-negative rate over the true negative rate, so that a decreasing likelihood ratio <1 indicated increasing probability the absence of PM. The positive likelihood ratio is defined as the true positive rate over the false-positive rate, so that an increasing likelihood ratio > 1 indicated increasing probability the diagnosis of PM. The confidence intervals show that the PM-CAD system demonstrates statistically better screening performance in terms of both negative likelihood ratio and positive likelihood ratio than radiologists. Radiologist 1 & 2: with < 5 years professional experience, Radiologist 3 & 4: with 5 - 10 years professional experience, Radiologist 5 & 6: with > 10 years professional experience. PM, pituitary microadenoma; receiver operating characteristics (ROC); the area under ROC curve (AUC). [file Image_5.TIF]

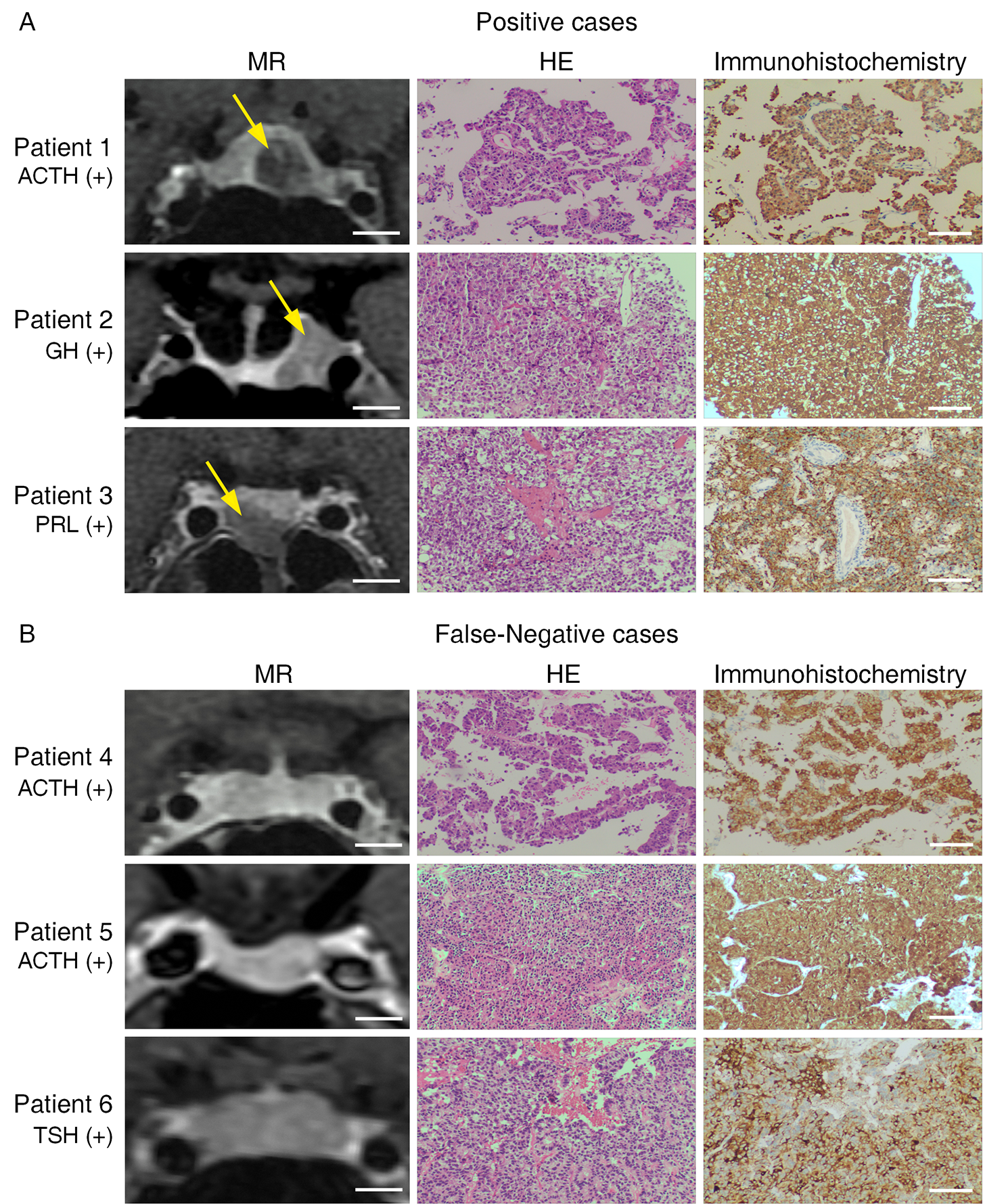

Supplement: Supplementary Figure 6 — The MRI and histological validation of double positive and false-negative cases. (A,B) 3 double positive and 3 false-negative cases, which were functional PM, as confirmed by subsequent pathological examination. The comprehensive clinical data for these patients are listed in Supplementary Table 5. PM, pituitary microadenoma; MRI, magnetic resonance imaging; AI, Artificial intelligence; HE, hematoxylin and eosin; ACTH, adrenocorticotropic hormone; GH, growth hormone; TSH, thyroid stimulating hormone; PRL, prolactin. MR bar = 5mm. Pathology bar =100 μm. The yellow arrow indicates a pituitary microadenoma. [file Image_6.TIF]

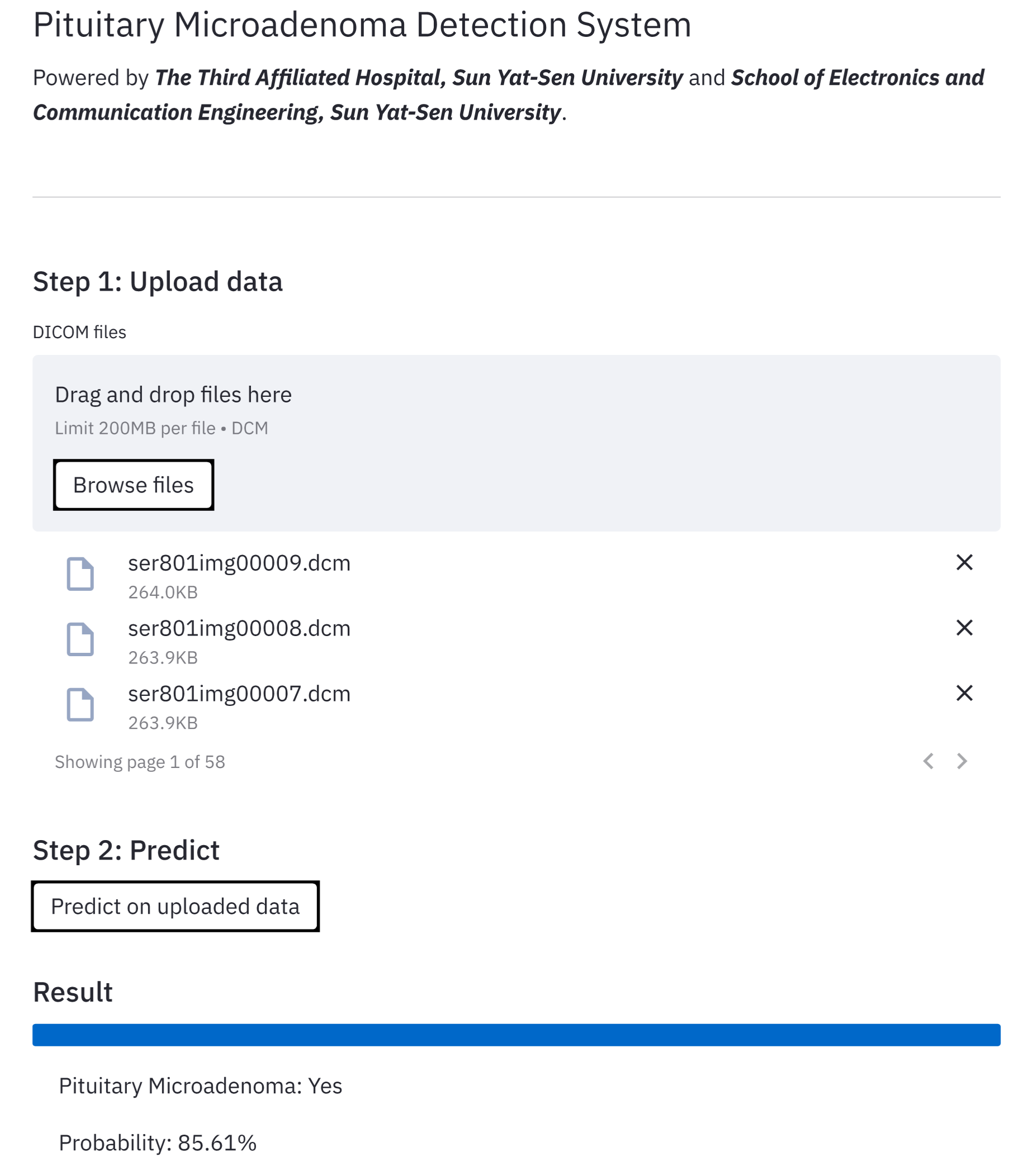

Supplement: Supplementary Figure 7 — The browser-based software to aid the diagnosis of PM. As long as we upload the pituitary MR images (DICOM), the software will tell you whether the patient suffering from PM disease. This browser based tool can be accessed at http://82.157.181.77/. [file Image_7.TIF]
